# Comparative efficacy of placebos in short-term antidepressant trials for major depression: a secondary meta-analysis of placebo-controlled trials

**DOI:** 10.1186/s12888-020-02839-y

**Published:** 2020-09-07

**Authors:** Lisa Holper, Michael P. Hengartner

**Affiliations:** 1 Department of Psychiatry, Psychotherapy, and Psychosomatics, University Hospital of Psychiatry, University of Zurich, Lenggstrasse 31, 8032 Zurich, Switzerland; 2 Department of Applied Psychology, Zurich University of Applied Sciences, Zurich, Switzerland

**Keywords:** Bayesian network meta-analysis, Placebo, Antidepressants, Unblinding, Side-effects

## Abstract

**Background:**

The issue of unblinded outcome-assessors and patients has repeatedly been stressed as a flaw in allegedly double-blind antidepressant trials. Unblinding bias can for example result from a drug‘s marked side effects. If such unblinding bias is present for a given drug, then it might be expected that the placebos of that drug are rated significantly less effective than that of other antidepressants.

**Methods:**

To test this hypothesis, the present exploratory analysis conducted a Bayesian network meta-analysis (NMA) comparing the efficacy of 19 different placebos in placebo-controlled trials provided in the dataset by Cipriani et al. (Lancet 2018; 391: 1357–66). Primary outcome was efficacy (continuous) estimated on the standardized mean difference (SMD) scale and defined as the pre-post change on the Hamilton Depression scale (HAMD-17), on which information was available in *N* = 258 trials.

**Results:**

Comparative placebo ranking suggested mirtazapine-placebo (SMD -2.0 [− 5.0–1.0 95% CrI]) to be the most, and amitriptyline- (SMD 1.2 [− 1.6–3.9 95% CrI]) and trazodone- (SMD 2.1 [− 0.9–5.2 95% CrI]) placebos to be the least effective placebos. Other placebos suggested to be more effective than amitriptyline- and trazodone-placebos (based on 95% CrIs excluding zero) were citalopram, desvenlafaxine, duloxetine, escitalopram, fluoxetine, sertraline, and venlafaxine placebos. These NMA results were corroborated by the observation that the relative efficacy between drug and placebo was considerably larger for amitriptyline and trazodone than for instance mirtazapine, duloxetine, and venlafaxine, supported by a small and insignificant correlation between drug-efficacy and placebo-efficacy (r = − 0.202, *p* = 0.408).

**Discussion:**

The present exploratory NMA indicates that distinguishable side effects of older drugs may unblind outcome-assessors thus resulting in overestimation of the average drug-placebo difference and underrating bias in placebo-arms, particularly for the older antidepressant drugs amitriptyline and trazodone. If confirmed in prospective studies, these findings suggest that efficacy rankings for antidepressants are susceptible to bias and should be considered unreliable or misleading. The analysis is limited by the focus on the single-comparison placebos (76%, i.e., placebos assessed in two-arm trials), since double-comparison placebos (25%, i.e., placebos assessed in three-arm trials) are hard to interpret and therefore not included in the present interpretation. Another limitation is the problem of multiplicity, which was only approximately accounted for in the Bayesian NMA by modelling treatment effects as exchangeable.

## Background

The controversy about the clinical benefits of new-generation antidepressants for the acute treatment of depression is ongoing and unresolved [1–4]. One major issue with antidepressant trials is that they exclusively rely on subjective outcomes, that is, clinician-ratings of depression symptoms. Almost all antidepressant trials are at high or unclear risk of bias with respect to allocation concealment and blinding of outcome-assessors [1], two important biases known to inflate effect size estimates for subjective outcomes [5, 6].

Although the issue of unblinded outcome-assessors and patients has repeatedly been stressed for years as a major flaw in allegedly double-blind psychiatric drug trials [7–9], it is poorly studied because the vast majority of trials does not assess (or report) unblinding [10]. Nevertheless, some studies have shown that in trials with older drugs such as tricyclic antidepressants (TCAs), e.g., amitriptyline, the blind was frequently broken due to the drugs‘ marked side effects like sedation, drowsiness, dizziness and dry mouth [11]. As early as in 1967, Leyburn [12] noted in a Lancet article that “most antidepressant drugs cause side-effects which are recognizable by experienced doctors in a significant proportion of patients. Patients who come into the consulting-room for assessments, perhaps for the sixth time and rather bored with the whole thing, but with their mouths so dry that one can hear their tongues scraping and clicking about in their mouths, are likely to be taking, say, amitriptyline, rather than the placebo”. That is, outcome-assessors in trials of older antidepressants were able to detect with high accuracy which trial participants received the active treatment and which placebo. This is a serious issue, because unblinding is associated with inflated response estimates for depression treatments [13, 14]. These findings are strongly supported by randomized trials of TCAs using active placebos that also cause anticholinergic side effects. In these truly blinded trials, the average drug-placebo difference was much smaller than in TCA trials with inert placebos [15]. It is thus plausible that TCAs appeared highly effective because outcome-raters were able to break blind and hence to correctly guess who was on active treatment and who on inert placebo. The same principle probably holds for trazodone, which is also an older sedating drug that can cause marked drowsiness and dizziness [16, 17] and that is poorly tolerated relative to selective serotonin reuptake inhibitors (SSRIs), serotonin–norepinephrine reuptake inhibitors (SNRIs) and other atypical new-generation antidepressants like mirtazapine and agomelatine [2].

If this assumption of unblinding bias with older (sedating) antidepressants is true, then we would expect that the placebos for older drugs are rated significantly less effective than those for newer antidepressants that are better tolerated and have less detectable side effects [2, 18]. Naudet and colleagues [19] previously conducted a meta-analysis that compared the response to different placebos. The analysis however included a small number of trials (*N* = 31) comparing only fluoxetine and venlafaxine, which did not reveal differences between fluoxetine- and venlafaxine-placebo. In the present analysis, we expand their work by focussing on all new-generation antidepressant-placebos in comparison to the placebos for the older drugs amitriptyline and trazodone. Based on the rationale detailed above, we hypothesized that due to unblinding of outcome-assessors the placebos of the older drugs would be rated less effective than the placebos of the newer drugs, which are more difficult to correctly guess due to their more favourable side effect profile.

## Methods

The exploratory analysis was not based on a written protocol, but followed the findings of Naudet and colleagues [19].

### Data sources

A total of 308 randomized placebo-controlled trials conducted between 1979 and 2016 (240 published studies, 68 unpublished studies) were identified. Three hundred four trials constituted all the placebo-controlled trials provided in the GRISELDA dataset by Cipriani and colleagues [2], and 4 trials were provided by Furukawa and colleagues [20]. The supplementary appendix provides a list of a included studies (Additional file 2). Our primary outcome was efficacy (continuous) estimated on the standardized mean difference (SMD) scale and defined as the pre-post change on the Hamilton Depression scale (HAMD-17) [21], on which information was available in *N* = 258 trials. All placebos were inactive placebos. A PRISMA flow-chart detailing the study selection process is given in the supplementary Fig. S1.

Together, the present dataset compared 19 antidepressants; agomelatine (AGO), amitriptyline (AMI), bupropion (BUP), citalopram (CIT), desvenlafaxine (DES), duloxetine (DUL), escitalopram (ESC), fluoxetine (FLO), fluvoxamine (FLU), levomilnacipran (LEV), mirtazapine (MIR), nefazodone (NEF), paroxetine (PAR), reboxetine (REB), sertraline (SER), trazodone (TRA), venlafaxine (VEN), vilazodone (VIL), and vortioxetine (VOR). Clomipramine (CLO) was not included because in the only placebo-controlled trial available any information about efficacy was missing. Milnacipran (MIL) was not included because no placebo-controlled trials were available.

**Fig. 1:**
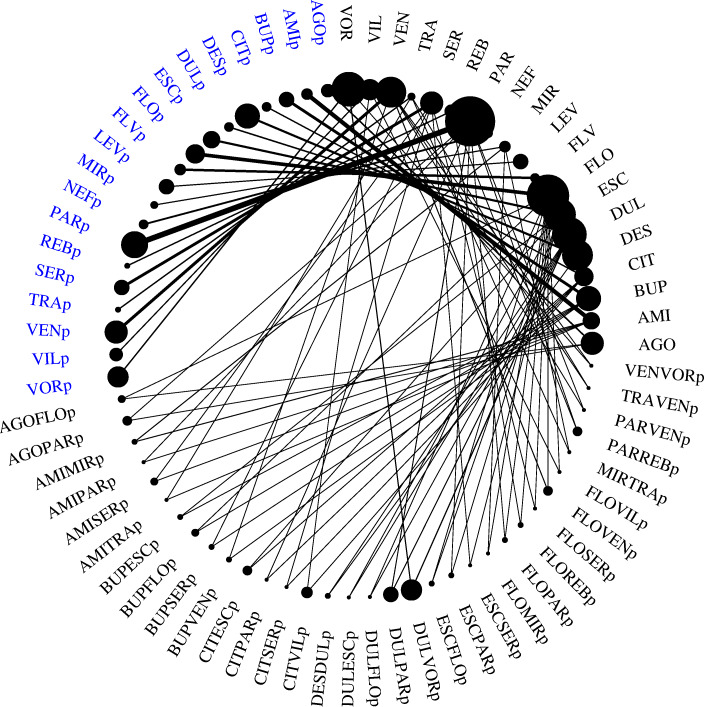
Network graph. Summary of the evidence of the network comparing drugs versus placebos. The thickness of the lines is proportionate to the number of trials comparing each pair of drugs/placebos, and the size of each node is proportionate to the number of randomized participants (sample size); see Fig. S2 for details on the original network and Tab. S1 for details on sample sizes. Single-comparison placebos discussed in the main analysis, i.e., those assessed in two-arm trials, are highlighted (blue)

The primary aim of the present analysis was to compare placebo arms. Placebos were therefore renamed according to the antidepressants to which they were compared with, appended by the letter ‘p’ (Fig. 1), following Naudet and colleagues [19]. Placebos that were compared to a single antidepressant (i.e., single-comparison placebos assessed in two-arm trials) were named after the one drug; and placebos that were compared to two antidepressants (i.e., double-comparison placebos assessed in three-arm trials) were named after both drugs. For example, placebos compared to amitriptyline (AMI) were named AMIp; and, placebos compared to amitriptyline (AMI) and mirtazapine (MIR) were named AMIMIRp. There were no four-arm trials comparing placebo against three different antidepressants; any existing four-arm trials merely considered different dosages and were therefore named according to three-arm trials. This resulted in *N* = 52 different placebos, 19 of which were single-comparison placebos (*N* = 193 trials) and 33 were double comparison placebos (*N* = 65 trials, supplementary Fig. S2, Tab. S1). The last placebo in alphabetical order (VENVORp) was chosen as reference.

### Unadjusted Bayesian NMA

Modeling was conducted based on standard Bayesian random-effects NMA [22], using the JAGS software (version 4·3·0) [23]. Simulations were run for 3 chains with an adaptive phase of 100′000, a burn-in of 100′000, and a sampling phase of 200′000 iterations, thinned such that every 10*th* iteration was retained. Convergence was ensured by considering the Brooks–Gelman–Rubin diagnostics [24] with the potential scale reduction factor $\overline{R}$ ≤ 1.05 accepted as implying convergence [25]. Bayesian model fit was based on the deviance information criterion (DIC), a measure of goodness of fit and complexity [25]. Our primary outcome efficacy (continuous) was estimated on the standardized mean difference (SMD) scale.

Multiplicity issues were accounted for by using a symmetric random-effects NMA model with exchangeable treatment effects [26], which have been shown to fit well when there is no obvious placebo or other reference treatment in the network, as it was the case in the present analysis.

Trial baselines were assumed to have exchangeable effects in order to account for disconnected treatments and placebos [27], which was the case for the drug-placebo comparisons FLV-FLVp, LEV-LEVp, and NEF-NEFp resulting from the afore-mentioned renaming of placebos.

### Covariate adjusted Bayesian NMA

Covariate adjusted sensitivity analysis was conducted to test the robustness of the main analysis by adjusting for the trial-level covariates, study center (multi- versus single-center), study dosing schedule (flexible versus fixed dose), study length (range 4–12 weeks), sample size, study year (continuous covariate), study year (categorical covariate, before versus after 2000), publication status (published versus unpublished trials), and sponsorship (sponsored versus unsponsored trials). The covariate study year was defined as study year of completion, study year of publication, or year of drug approval from the FDA (US Food and Drug Administration), where available in this order [20]; preference was given to study year of completion, because unpublished trials, by definition, have no year of publication. The resulting study year range was 1979–2014. Treatment-by-covariate and placebo-by-covariate interactions were assumed to be exchangeable-related drawn from a random distribution with common mean (*B*) and between-treatment variance (σ_*B*_) [28]. The supplementary appendix provides details on the methods applied.

## Results

The exploratory results presented in the main text focus on the single-comparison placebos only, since the double-comparison placebos are hard to interpret due to the relative influence of two antidepressants (Fig. 1). The supplementary appendix provides details on all placebos.

In accordance with previous re-analyses of the Cipriani dataset, all placebos were less effective than antidepressants (95% credible intervals [CrIs] excluding zero) [1, 3, 4], in line with the main results reported by Cipriani and colleagues [2].

**Fig. 2:**
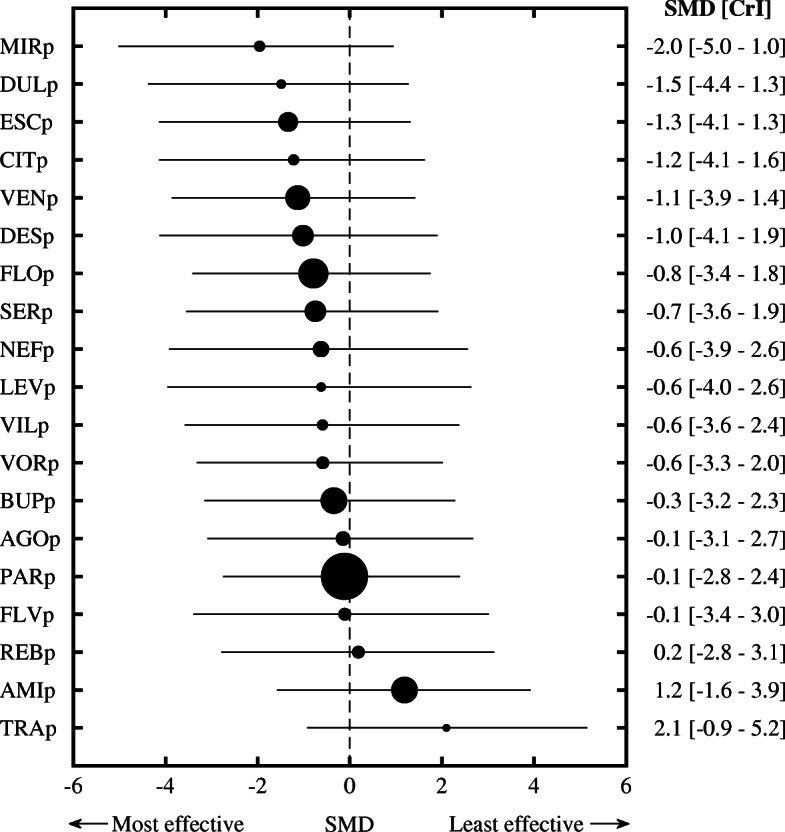
Forest plot. Forest plot comparing placebo efficacy estimates on the standardized mean difference (SMD) scale with credible intervals [CrI]. Circle size is proportionate to trial size. The last placebo in alphabetical order (VENVORp) was chosen as reference placebo, which is thus set to zero in this plot. Placebos are ranked from most to least effective (top to bottom). See Fig. S3 for details on all placebos

**Fig. 3:**
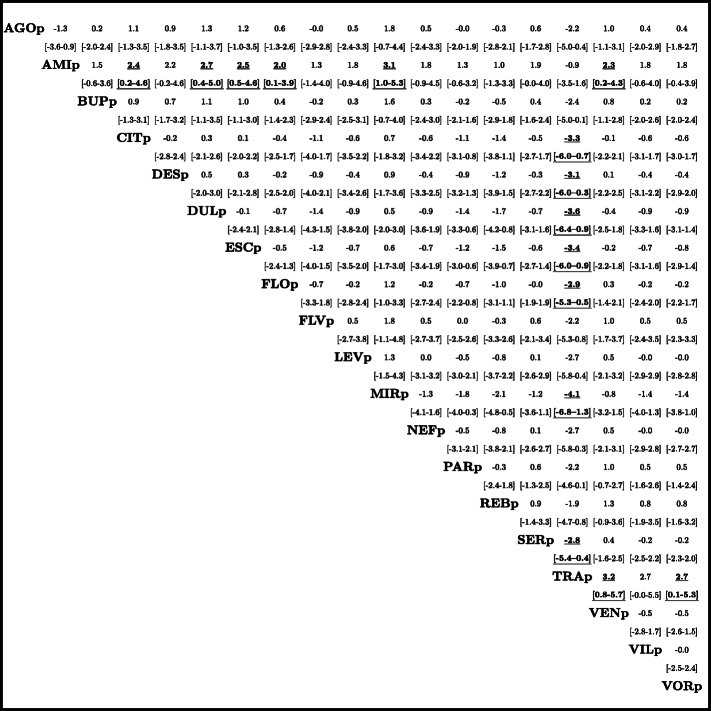
Pairwise comparisons. Listed are pairwise comparisons between placebos efficacy (standardized mean difference, SMD) with 95% credible intervals [CrI]. SMD smaller than 0 favor the row-defining placebo, and vice versa, SMD larger than 0 favor the column-defining placebo. Significant results are bold and underscored. See Fig. S4 and Tab. S2 for details on all placebos

Comparative ranking of the placebos suggested mirtazapine-placebo (MIRp, SMD -2.0 [− 5.0–1.0 95% CrI]) to be the most effective placebo, whereas amitriptyline- (AMIp, SMD 1.2 [− 1.6–3.9 95% CrI]) and trazodone- (TRAp, SMD 2.1 [− 0.9–5.2 95% CrI]) placebos were suggested to be the least effective placebos (Fig. 2, supplementary Fig. S3). In particular, placebos suggested to be more effective than AMIp (based on 95% CrIs excluding zero) were citalopram (CITp), duloxetine (DULp), escitalopram (ESCp), fluoxetine (FLOp), mirtazapine (MIRp), and venlafaxine (VENp) placebos; and placebos suggested to be more effective than TRAp (based on 95% CrIs excluding zero) were citalopram (CITp), desvenlafaxine (DESp), duloxetine (DULp), escitalopram (ESCp), fluoxetine (FLOp), mirtazapine (MIRp), and sertraline (SERp) placebos (Fig. 3; supplementary Fig. S4, Tab. S2).

**Fig. 4:**
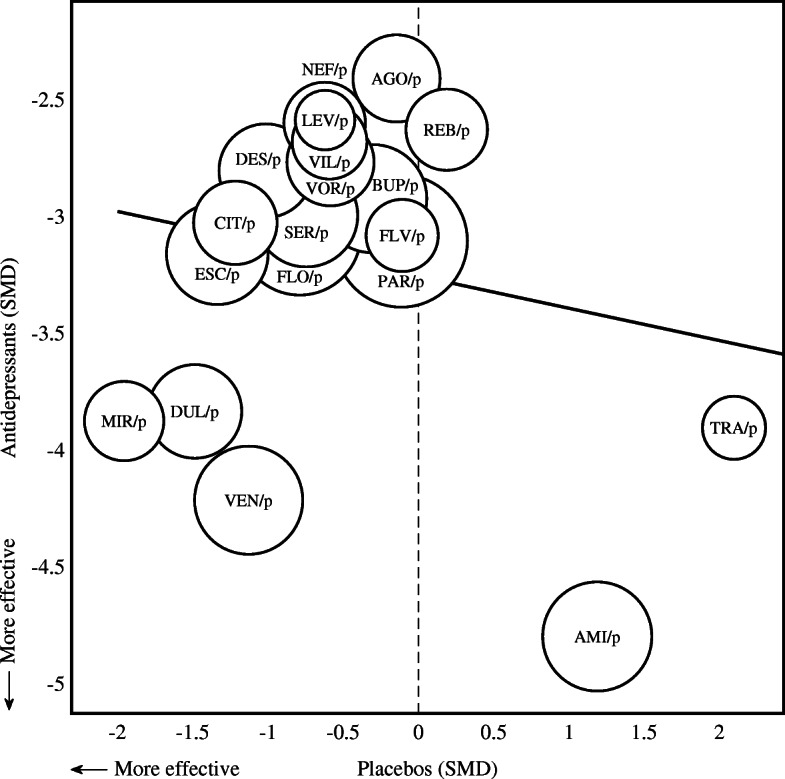
Correlation between drug-efficacy and placebo-efficacy. Scatter plot illustrating the correlation between drug and placebo efficacy on the standardized mean difference (SMD) scale. Circle size is proportionate to mean trial size (logarithm). Zero indicates no effect compared to the reference (VENVORp). The Pearson correlation was small and insignificant (r = − 0.202, *p* = 0.408)

Further, in order to corroborate our hypothesis that due to unblinding of outcome-assessors the placebos of the older drugs may be rated less effective than the placebos of the newer drugs, the correlation between drug-efficacy and placebo-efficacy was assessed. We observed that the overall correlation was small and statistically insignificant (r = − 0.202, *p* = 0.408). Consistent with our hypothesis, the differences in relative efficacy between drug and placebo was considerably larger for AMI/AMIp and TRA/TRAp (that is, negatively correlated) than for instance for MIR/MIRp, DUL/DULp, and VEN/VENp (Fig. 4).

Sensitivity analysis revealed that the differences between AMIp/TRAp and newer-generation antidepressant became weaker but remained largely unaltered after adjusting for the trial-level covariates study center, dosing schedule, study length, study size, study year (both adjusting for the continuous and categorical covariate), publication status, or sponsorship (supplementary Fig. S5, Tab. S3). Particularly, the difference between MIRp and AMIp, and all differences with TRAp remained significant (95% CrIs excluding zero).

## Discussion

In this secondary exploratory meta-analysis of the Cipriani dataset we tested whether the placebos of newer antidepressants were more effective than the placebos of the older drugs amitriptyline and trazodone. These two drugs, together with clomipramine, have been shown to be less well tolerated than the newer-generation antidepressants [2, 18]. Based on the unblinding of investigators documented in various studies [8, 10, 11], we therefore hypothesized that outcome-assessors in trials of these older drugs were more frequently unblinded due the drugs’ marked and observable side effects. By consequence, we assumed that the unblinded outcome-assessors would, consciously or unconsciously, underrate the response to placebos for these older drugs. In line with our reasoning, we found that the amitriptyline- and trazodone-placebos were rated less effective than the placebos of the newer, better tolerated, antidepressants, such as SSRIs (citalopram, escitalopram, fluoxetine, sertraline), SNRIs (duloxetine, desvenlafaxine, venlafaxine), and in particular the atypical noradrenergic and specific serotonergic antidepressant (NaSSA) mirtazapine**.** Because trial methodology, sample characteristics and the rate of positive trials have considerably changed over time [29, 30], we also controlled for important covariates such as study center, dosing schedule, study length, sample size, study year, publication status and sponsorship. Although the inferiority of the amitriptyline-placebo did not remain significant (95% CrIs including zero, notwithstanding the fact that it still indicated lower response) except in relation to mirtazapine-placebo, the differences for the trazodone-placebo compared to new-generation-placebos remained significant (95% CrIs excluding zero).

Our findings are compatible with the hypothesis that, due to unblinding, outcome-assessors may have overestimated the average drug-placebo difference for the older antidepressant drugs amitriptyline and trazodone. Other studies also support the view that unblinding may drive exaggerated response ratings for antidepressants relative to placebo. For instance, Khan and colleagues [14] found that the average response to depression treatments was higher when outcome-assessors were unblinded. The meta-analysis by Moncrieff and colleagues [15] found that the response to TCAs was poor when compared to active placebos (d = 0.17). Likewise, a meta-analysis by Greenberg and colleagues [13] found that the clinician-rated response to TCAs was small (d = 0.25) in “blinder” three-arm trials which contained an active-control in addition to placebo-control. Moreover, in these three-arm trials the response to the TCAs was close to zero (d = 0.06) when assessed with patient self-reports, suggesting that outcome-assessors see drug-placebo differences that the thus rated patients personally do not perceive.

The present findings are important for the interpretation of the comparative response to different antidepressants as provided by Cipriani and colleagues [2]. In their supplement, Cipriani and colleagues [2] reported that adjusting for the probability of receiving placebo increased the response to amitriptyline from OR = 2.13 to a striking OR = 3.16 (48% increase). Similarly, for trazodone, this resulted in an increase from OR = 1.51 to OR = 1.97 (30% increase). These findings clearly illustrate that the average treatment response for both amitriptyline and trazodone increases substantially when they were compared to placebo in a two-arm trial, presumably because including a placebo-arm makes it much easier for outcome-assessors to detect which participants received the investigational drug than in an active-controlled trial.

Consistent with our hypothesis that unblinding of outcome-assessors in trials of older drugs biases the average drug-placebo difference, a meta-analysis [31] of the placebo response has shown that the average placebo response in 2005 was more than twice larger than the placebo response in 1980 when assessed by outcome-assessors. However, no change over time was found for patient self-ratings [31], which again bolsters our findings detailed above that outcome-assessors rate drug-placebo differences differently to what patients personally perceive [13]. It is also important to stress that while the placebo response has considerably increased during the 1980s [32], since about 1991 the average placebo response remained largely constant around 35–40% when changes in trial design features are taken into account [20, 33].

We see no reason to assume that there is no unblinding in trials of SSRI, SNRI, or NaSSA antidepressants, although the bias is presumably less pronounced as the newer drugs are better tolerated than TCAs [18]. For example, mirtazapine, which has a unique dual mode of action as a noradrenergic and specific serotonergic antidepressant [34], has sedating effects due to its affinity to histamine receptors at low plasma concentrations [35]. This antihistamine effect, however, is offset at higher doses by increased noradrenergic transmission, which reduces its sedating effect [36–38]. Mirtazapine is further considered to have a lower risk of anticholinergic or serotonin-related adverse effects often associated with other antidepressants (such as sexual dysfunction, nausea, etc.), even lower than SSRIs, and may actually improve certain side effects when taken in conjunction with other antidepressants [39–41].

Nevertheless, new-generation antidepressants also cause side effects [42], which is why dropout rates due to adverse events are higher for new-generation antidepressants than placebo (but of course still lower than dropout rates of older antidepressants) [2]. Experienced clinicians may thus still be able to correctly guess, whether a participant receives placebo or active treatment. In accordance, in the re-analysis of the Hypericum Depression Trial, Chen et al. [43] showed that clinicians were better at correctly guessing placebo than sertraline or hypericum. In addition, side effects were more pronounced among participants for which the clinicians guessed active treatment (which indicates unblinding due to side effects), and improvements on active treatment relative to placebo were larger when the clinicians guessed active treatment. We therefore suggest that unblinding bias is also an issue in trials of newer antidepressants, although it is probably less pronounced than in trials of the poorer tolerated older antidepressants.

Finally, it is important to note that our analysis cannot fully rule out alternative explanations. For instance, instead of unblinding, another reason could be the transformation of trial protocols over time. To name just one example, inclusion and exclusion criteria of antidepressant trials have become more restrictive over time, meaning that trial participants are increasingly unrepresentative [44]. Although controlling for study year certainly reduces this confounding effect in part, it cannot remove it altogether. To confirm our hypothesis, a preregistered prospective study is required. Given that side effects that are observable for an outcome assessor even when not reported by the patient (e.g., dry mouth, tremor, drowsiness, somnolence) are presumably those causing unblinding, it would be worthwhile to examine whether these specific side effects (relative to less detectable side effects such as sexual dysfunction and lack of appetite) lead to correct identification of treatment received and whether they are negatively correlated with depression ratings in the placebo arm.

The main implication of our study is that unblinding should be systematically assessed and reported in antidepressant trials. This would allow to statistically control for unblinding effects and it would also be possible to conduct a confirmatory study as detailed above. If our hypothesis holds, it would imply that inert placebos are a poor control and thus the use of active placebos should be reconsidered. Another implication would be that efficacy rankings based on NMA must be interpreted with caution.

### Limitations

A limitation of the present analysis is that it was not based on a written protocol, but merely followed the findings of Naudet and colleagues [19].

Another limitation inherent in the present data set is that the placebos can only be interpreted based on their comparisons with the corresponding antidepressants to which they are bound in the network. Here, we focused on the single-comparison placebos, since the double-comparison placebos are hard to interpret and therefore only presented in the supplement. It should therefore be kept in mind that 24% of the trials also including double-comparisons were not included in the present interpretation.

Anther limitation concerns the evidence summarized in this special placebo NMA, in that all comparisons *between* placebos rely on indirect evidence only, and not on a mixture of direct and indirect comparisons as for most of the antidepressants; though, in mixed treatment comparisons, a main part of the evidence is also often based on indirect evidence [45]. The consistency hypothesis, assuming that effects between direct and indirect comparisons are the same, can therefore not be verified. Though, it is impossible in this placebo-context to verify this hypothesis, one cannot be sure of the validity of the comparisons considering that indirect comparisons may not be robust and prone to vibration of effects [46].

A methodological limitation is the problem of multiplicity in the present NMA. Standard NMA models usually do not account for multiple comparisons in estimating relative treatment effects, which might lead to exaggerated and overconfident statements regarding relative treatment effects. The present analysis therefore applied the Bayesian approximation to reduce that problem described by Efthimiou and White [26], where treatment effects are modelled exchangeable, and hence estimates are shrunk away from large values.

A more general limitation is that the reliance on the similarity hypothesis that assumes that all trials are similar enough to be pooled together. Cipriani et al. [2] considered this hypothesis to be valid, but still some unmeasured characteristics might have influenced our findings, such as differences between in- and outpatients or any other surrogate of depression severity at study entry.

## Conclusion

Considering clinician-rated symptom change, the present analysis suggests amitriptyline-placebo and in particular trazodone-placebo to be less effective than various SSRI- (citalopram, escitalopram, fluoxetine, sertraline), SNRI- (duloxetine, desvenlafaxine, venlafaxine), and NaSSA- (mirtazapine) placebos. A likely explanation might be that the distinguishable sedative side effects and poorer tolerability of amitriptyline and trazodone may have resulted in unblinding of outcome-assessors and consequently in an overestimation of the average drug-placebo difference and an underrating of symptom-change in the placebo-arms. These findings illustrate that efficacy rankings for antidepressants are susceptible to bias and thus may be considered unreliable or even misleading. Unless proven otherwise, it may be assumed that the blind is regularly broken in antidepressant trials when drugs have marked and distinguishable side effect profiles. However, our exploratory post-hoc analysis cannot rule out alternative explanations, which is why the influence of side effects on unblinding should be tested in preregistered confirmatory studies.

## Supplementary information

**Additional file 1.**

**Additional file 2.**

## Data Availability

All results from this research are available in supplementary appendix. The GRISELDA dataset by Cipriani and colleagues [2] can be found in the Mendeley data repository: https://data.mendeley.com/datasets/83rthbp8ys/2

## Abbreviations

AGO: Agomelatine
AMI: Amitriptyline
NaSSA: Atypical noradrenergic and specific serotonergic antidepressant
BUP: Bupropion
CIT: Citalopram
CrI: Credible interval
CLO: Clomipramine
DES: Desvenlafaxine
DIC: Deviance information criterion
DUL: Duloxetine
ESC: Escitalopram
FLO: Fluoxetine
FLU: Fluvoxamine
HAMD-17: Hamilton depression scale
LEV: Levomilnacipran
MIL: Milnacipran
MIR: Mirtazapine
NEF: Nefazodone
NMA: Network meta-analysis
PAR: Paroxetine
REB: Reboxetine
SSRIs: Selective serotonin reuptake inhibitors
SNRIs: Serotonin–norepinephrine reuptake inhibitors
SER: Sertraline
SMD: Standardized mean difference
TRA: Trazodone
TCAs: Tricyclic antidepressants
VEN: Venlafaxine
VIL: Vilazodone
VOR: Vortioxetine
