# Supplementary material for: Comparative efficacy of placebos in short-term antidepressant trials for major depression: a secondary meta-analysis of placebo-controlled trials
Source: BMC Psychiatry. 2020 Sep 7;20:437. doi: 10.1186/s12888-020-02839-y (PMC7487933; doi:10.1186/s12888-020-02839-y)
Supplement: Supplementary file 1 — Additional file 1. [file 12888_2020_2839_MOESM1_ESM.pdf]

Supplementary appendix: Comparative response of placebos in short-term antidepressant trials for major depression: A secondary meta-analysis of placebo-controlled trials

Holper L, Department of Psychiatry, Psychotherapy, and Psychosomatics, University Hospital of Psychiatry, University of Zurich, Switzerland

Hengartner MP, Section for Clinical Psychology and Health Psychology, Zurich University of Applied Sciences/ZHAW, Zurich, Switzerland

Contents

1 PRISMA flow diagram **3**

2 Network graphs **5**

3 NMA unadjusted model **8**

4 NMA covariate adjusted models **11**

5 Code models **16**

5.1 Code unadjusted model 16

5.2 Code covariate adjusted model 17

References **20**

List of Figures

1 **PRISMA flow diagram.** 3

2a **Network graph original.** 5

2b **Network graph placebos.** 6

3 **Forest plot.** 8

4 **Pairwise comparisons.** 9

5 **Covariate pairwise comparisons.** 15

List of Tables

1 **Excluded drugs due to missing information on efficacy outcome.** . . . 4

2 **Trial and sample sizes of drugs and placebos.** 7

3 **Pairwise comparisons.** 10

4a **Covariate statistics.** 13

4b **Covariate estimates.** 14

1 PRISMA flow diagram

Figure S 1: PRISMA flow diagram. PRISMA flow chart illustrating the data selection process. See **Table S1** for details on excluded drugs due to missing information on efficacy outcome.

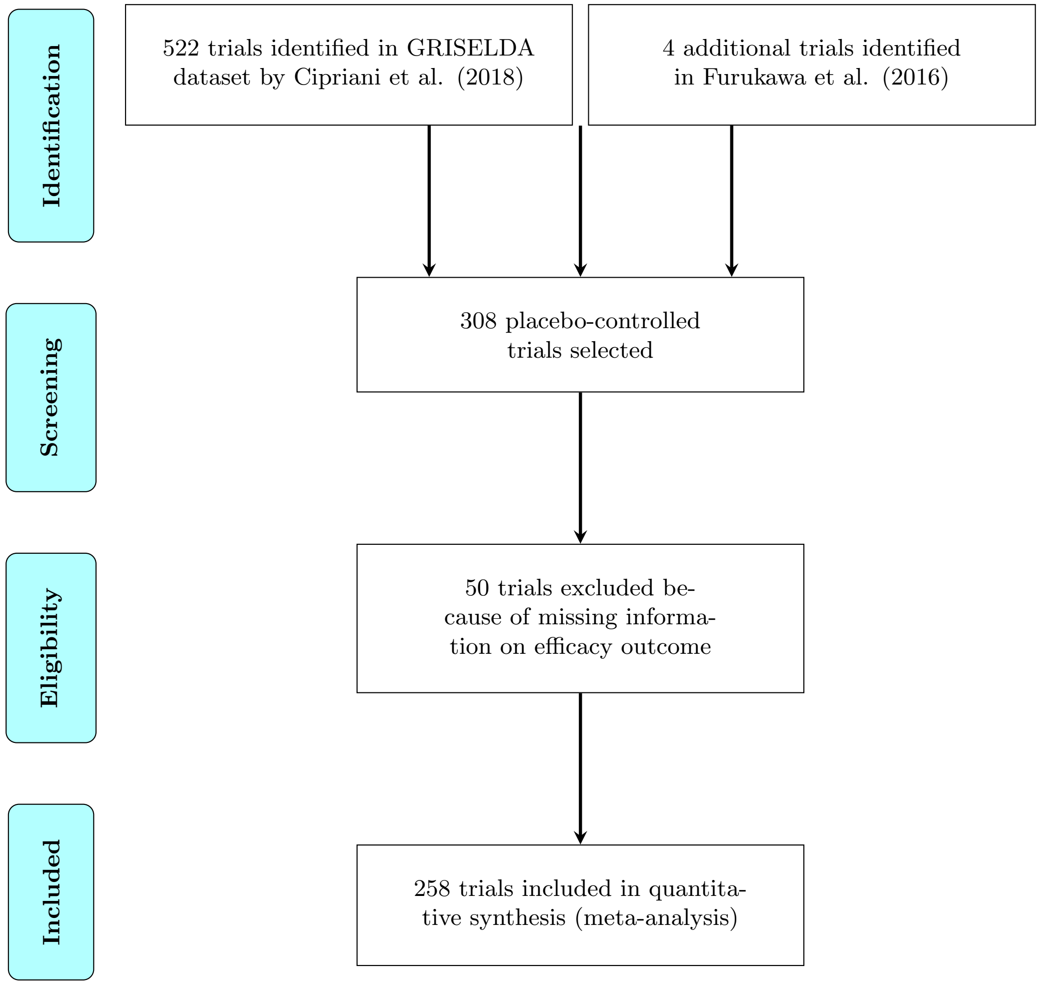

Table S 1: Excluded drugs due to missing information on efficacy outcome. Listed are excluded drugs due to missing information on efficacy outcome (see **Figure S1**).

**Drugs/placebos**	**Number**	**Percentage (%)**
AMI	23	10.6
AMIMIRp	1	0.5
AMITRAp	2	0.9
AMIVENp	3	1.4
AMIp	18	8.3
BUPp	2	0.9
CIT	2	0.9
CITp	2	0.9
CLO	2	0.9
CLOp	2	0.9
DUL	4	1.8
DULp	4	1.8
ESC	2	0.9
ESCp	2	0.9
FLO	10	4.6
FLONEFp	3	1.4
FLOp	7	3.2
FLV	18	8.3
FLVp	14	6.5
LEV	2	0.9
LEVp	2	0.9
MIR	3	1.4
NEF	8	3.7
NEFp	5	2.3
PAR	12	5.5
PARp	12	5.5
SER	16	7.4
SERp	8	3.7
TRA	8	3.7
TRAp	6	2.8
VEN	8	3.7
VENp	6	2.8

2 Network graphs

Figure S 2a: Network graph original. Summary of the evidence in the original network comparing drugs versus placebo. The thickness of the lines is proportional to the number of trials comparing each pair of treatments, and the size of each node is proportional to the number of randomized participants (sample size). Details on the number of trials are provided in **Tab. S1**.

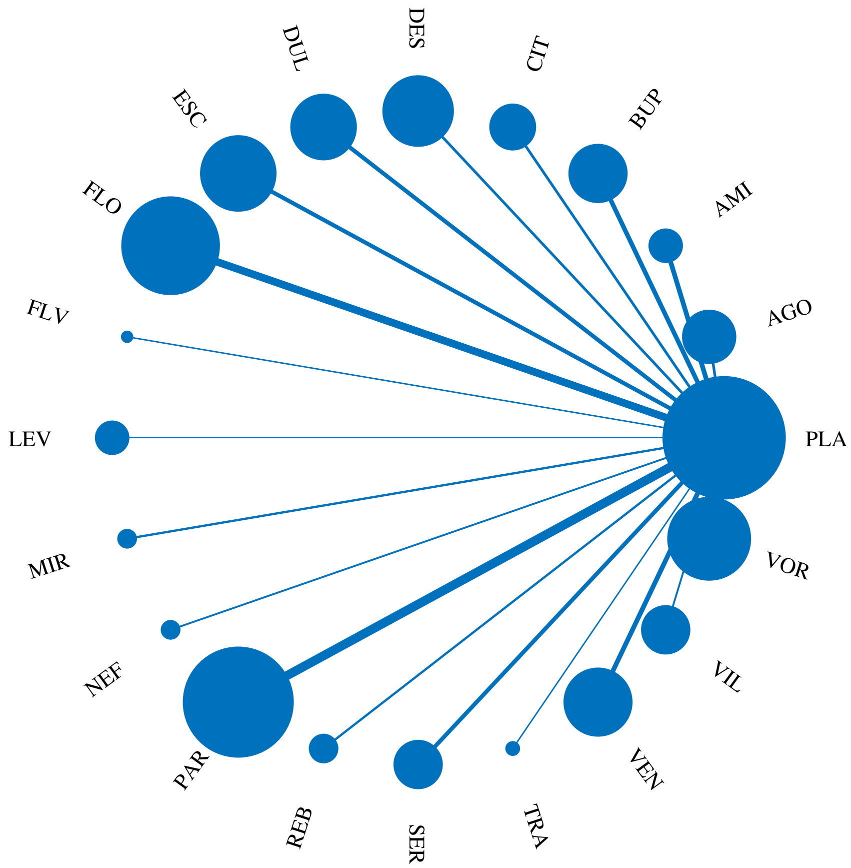

Figure S 2b: Network graph placebos. Summary of the evidence of the network used in the present analysis comparing drugs versus different placebos. The thickness of the lines is proportional to the number of trials comparing each pair of treatments, and the size of each node is proportional to the number of randomized participants (sample size). Disconnected drugs/placebos are highlighted (red). Details on the number of trials are provided in **Tab. S1**.

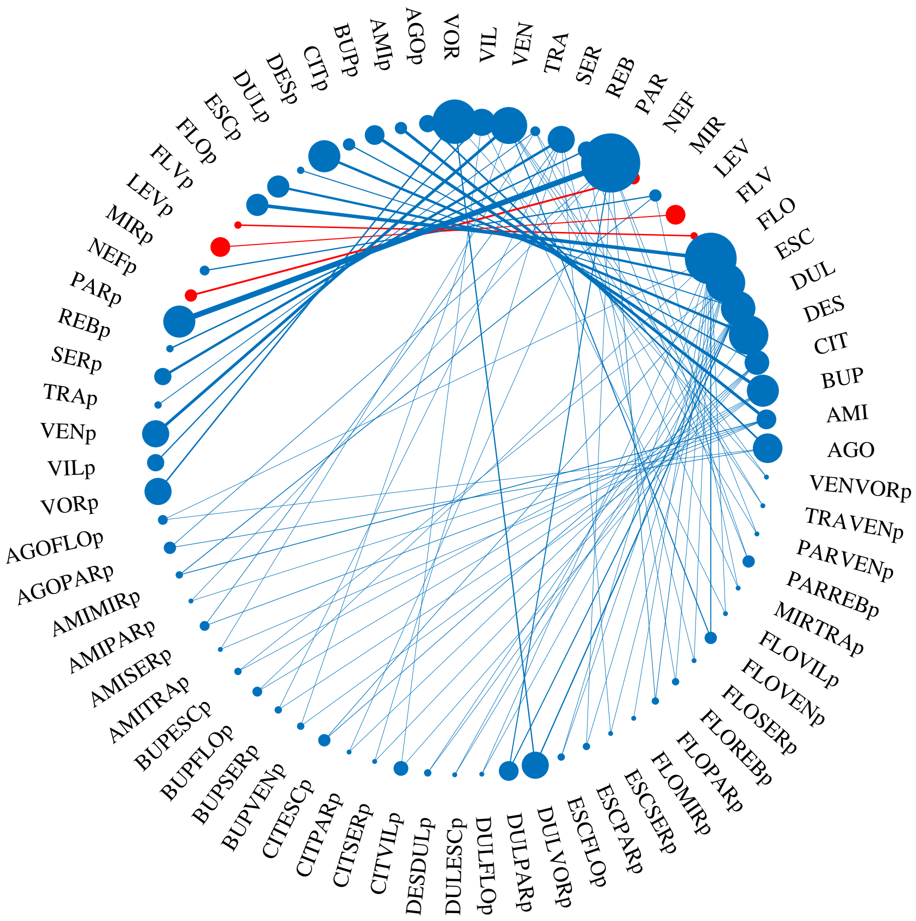

Table S 2: Trial and sample sizes of drugs and placebos. Listed are numbers of trials (T) and sample sizes (S) for drugs and placebos.

Drugs			Single-comparison Placebos			Double-comparison Placebos		
	Trials	Sample size		Trials	Sample size		Trials	Sample size
AGO	13	2324	AGOp	8	956	AGOFLOp	2	307
AMI	23	1543	AMIp	15	865	AGOPARp	3	442
BUP	22	2822	BUPp	15	1423	AMIMIRp	4	193
CIT	13	2586	CITp	6	596	AMIPARp	1	12
DES	13	3233	DESp	12	2051	AMISERp	3	359
DUL	20	3245	DULp	5	566	AMITRAp	1	68
ESC	20	3292	ESCp	11	1410	BUPESCp	2	278
FLO	37	4919	FLOp	17	1414	BUPFLOp	2	306
FLV	7	518	FLVp	7	473	BUPSERp	2	245
LEV	5	1603	LEVp	5	1051	BUPVENp	1	199
MIR	11	874	MIRp	6	317	CITESCp	3	410
NEF	9	809	NEFp	9	553	CITPARp	1	105
PAR	44	5939	PARp	27	2092	CITSERp	1	108
REB	11	1361	REBp	7	467	CITVILp	2	419
SER	20	2289	SERp	12	1157	DESDULp	1	164
TRA	7	621	TRAp	4	416	DULESCp	1	137
VEN	23	3266	VENp	14	1495	DULFLOp	2	145
VIL	9	1832	VILp	6	1196	DULPARp	5	517
VOR	14	3371	VORp	7	1231	DULVORp	6	963
						ESCFLOp	1	180
						ESCPARp	1	124
						ESCSERp	1	135
						FLOMIRp	1	72
						FLOPARp	3	277
						FLOREBp	2	278
						FLOSERp	1	105
						FLOVENp	5	510
						FLOVILp	1	99
						MIRTRAp	1	50
						PARREBp	2	511
						PARVENp	1	83
						TRAVENp	1	76
						VENVORp	1	105

3 NMA unadjusted model

Figure S 3: Forest plot. Forest plot illustrating drugs and placebos (standardized mean difference, SMD). Circle size is proportional to trial size. The last placebo in alphabetical order (VENVORp) was chosen as reference treatment.

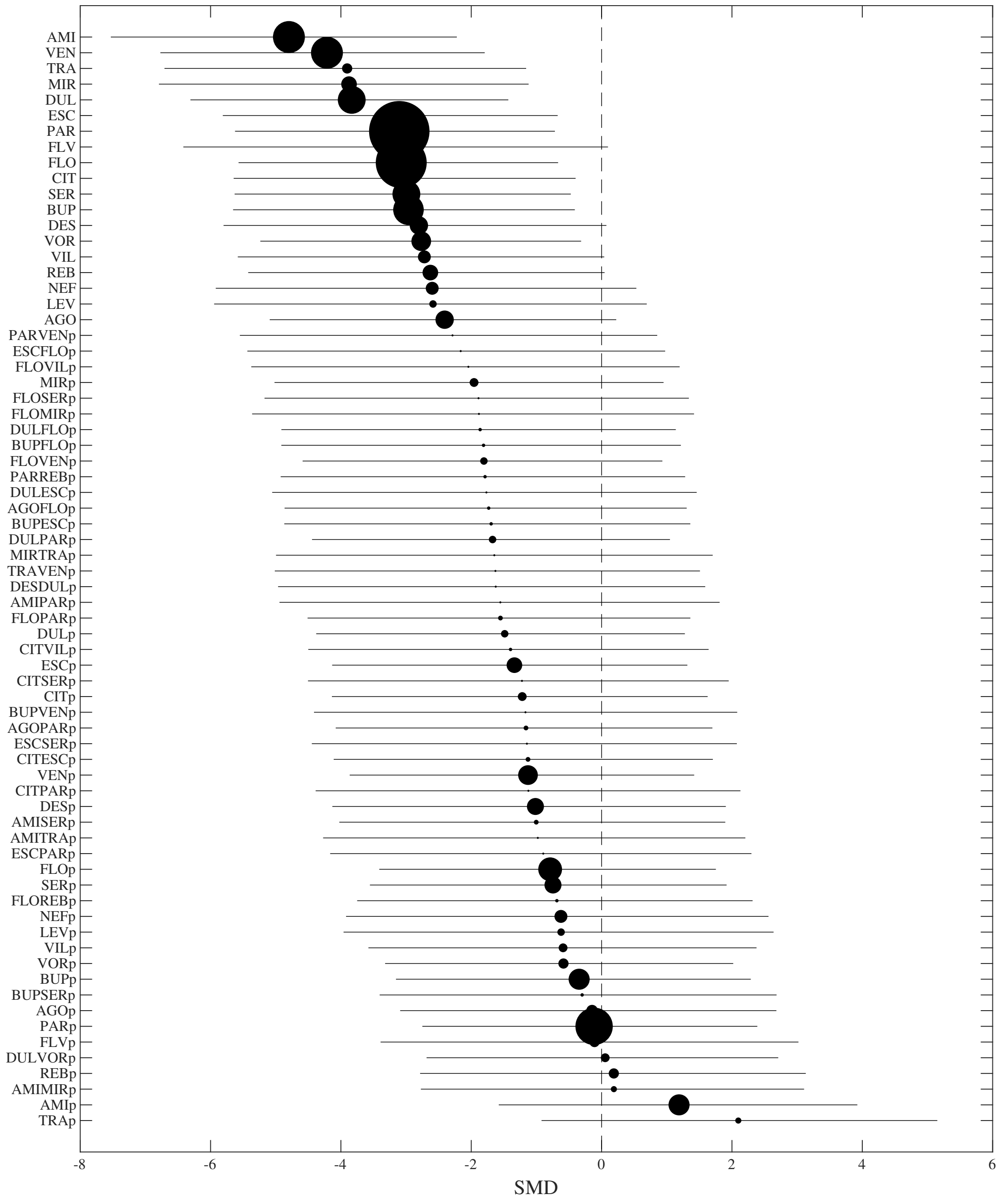

Figure S 4: Pairwise comparisons. Plot illustrating pairwise comparisons between drugs and placebos. Comparisons with credible intervals (CrIs) excluding zero are highlighted; blue color favors the treatment indicated in the row, and vice versa, red color favors the treatment indicated in the column; see **Tab. S2** for details.

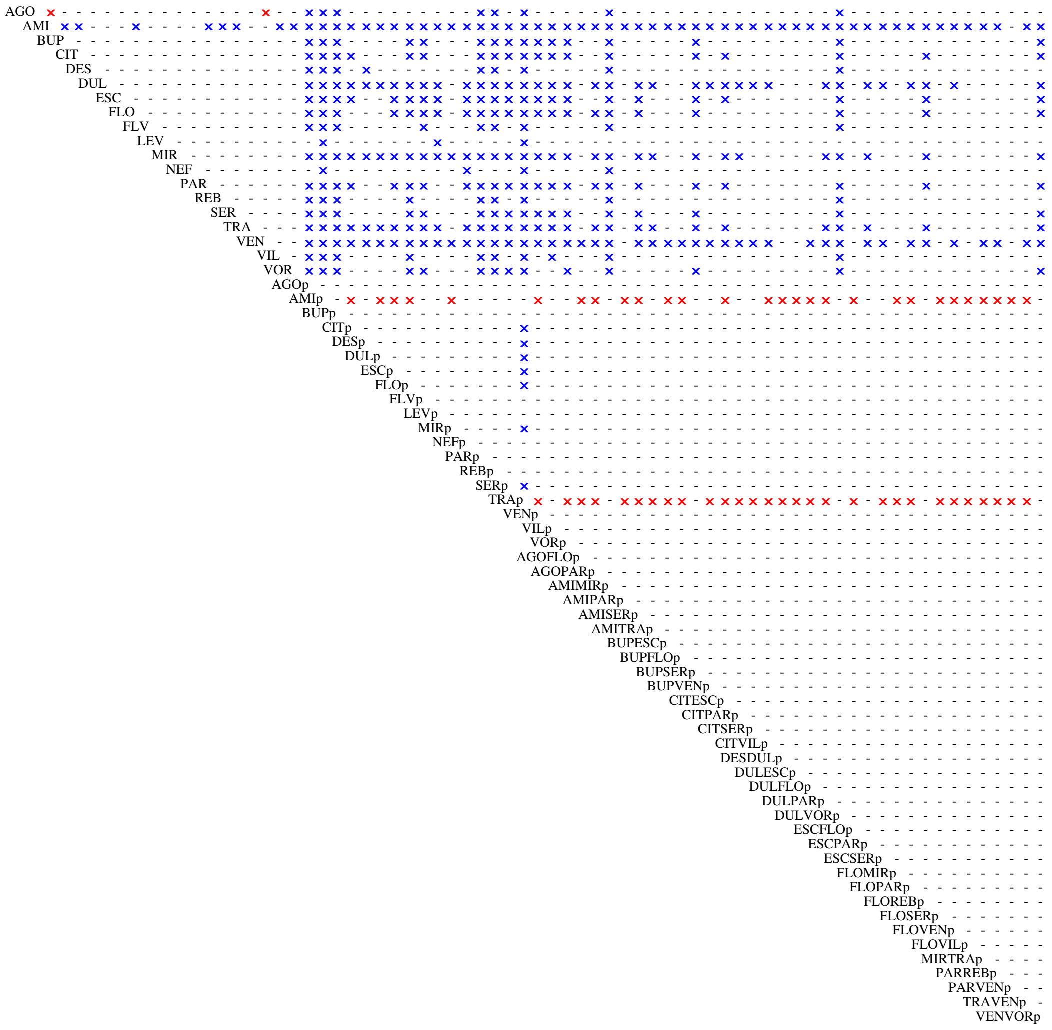

Table S 3: Pairwise comparisons. Listed are pairwise comparisons between drugs and placebos (standardized mean difference, SMD, with credible intervals, CrI). SMD smaller than 0 favor the row-defining drug or placebo, and vice versa, SMD larger than 0 favor the column-defining drug or placebo. Significant results are highlighted. For brevity, only comparison between placebos are shown; see **Fig. S4** for details.

AGOp	-1.3 [-3.6-0.9]	0.2 [-2.0-2.4]	1.1 [-1.3-3.5]	0.9 [-1.8-3.5]	1.3 [-1.1-3.7]	1.2 [-1.0-3.5]	0.6 [-1.3-2.6]	-0.0 [-2.9-2.8]	0.5 [-2.4-3.3]	1.8 [-0.7-4.4]	0.5 [-2.4-3.3]	-0.0 [-2.0-1.9]	-0.3 [-2.8-2.1]	0.6 [-1.7-2.8]	-2.2 [-5.0-0.4]	1.0 [-1.1-3.1]	0.4 [-2.0-2.9]	0.4 [-1.8-2.7]	1.6 [-0.8-4.0]	1.0 [-1.2-3.2]	-0.3 [-2.8-2.1]	1.4 [-1.5-4.4]	0.9 [-1.6-3.3]	0.8 [-2.1-3.7]	1.5 [-1.1-4.2]	1.7 [-0.8-4.3]	0.2 [-2.5-2.8]	1.0 [-1.9-3.9]	1.0 [-1.5-3.4]	1.0 [-1.8-3.8]	1.1 [-1.9-3.9]	1.3 [-1.4-3.9]	1.5 [-1.4-4.3]	1.6 [-1.3-4.5]	1.7 [-0.8-4.3]	1.5 [-0.7-3.7]	-0.2 [-2.5-2.0]	2.0 [-0.8-4.9]	0.7 [-2.1-3.6]	1.0 [-1.9-3.9]	1.7 [-1.3-4.7]	1.4 [-1.0-3.8]	0.5 [-2.0-3.1]	1.7 [-1.1-4.6]	1.7 [-0.6-4.0]	1.9 [-0.9-4.8]	1.5 [-1.4-4.4]	1.6 [-0.9-4.2]	2.1 [-0.7-4.9]	1.5 [-1.4-4.4]	-0.1 [-3.1-2.7]
AMIp		1.5 [-0.6-3.6]	2.4 [0.2-4.6]	2.2 [-0.2-4.6]	2.7 [0.4-5.0]	2.5 [0.5-4.6]	2.0 [0.1-3.9]	1.3 [-1.4-4.0]	1.8 [-0.9-4.6]	3.1 [1.0-5.3]	1.8 [-0.9-4.5]	1.3 [-0.6-3.2]	1.0 [-1.3-3.3]	1.9 [-0.0-4.0]	-0.9 [-3.5-1.6]	2.3 [0.2-4.3]	1.8 [-0.6-4.0]	1.8 [-0.4-3.9]	2.9 [0.4-5.4]	2.3 [0.0-4.7]	1.0 [-0.9-2.9]	2.7 [0.1-5.5]	2.2 [0.1-4.3]	2.2 [-0.4-4.9]	2.9 [0.3-5.4]	3.0 [0.5-5.5]	1.5 [-1.0-3.9]	2.4 [-0.4-5.1]	2.3 [0.0-4.6]	2.3 [-0.3-5.0]	2.4 [-0.4-5.3]	2.6 [0.1-5.1]	2.8 [0.1-5.6]	3.0 [0.2-5.8]	3.1 [0.6-5.5]	2.9 [0.7-4.9]	1.1 [-1.0-3.3]	3.4 [0.6-6.1]	2.1 [-0.7-4.8]	2.3 [-0.4-5.1]	3.1 [0.2-6.0]	2.7 [0.4-5.1]	1.9 [-0.6-4.3]	3.1 [0.3-5.8]	3.0 [0.8-5.2]	3.2 [0.5-6.0]	2.8 [0.1-5.6]	3.0 [0.5-5.6]	3.5 [0.8-6.2]	2.8 [0.1-5.6]	1.2 [-1.6-3.9]
BUPp			0.9 [-1.3-3.1]	0.7 [-1.7-3.2]	1.1 [-1.1-3.5]	1.0 [-1.1-3.0]	0.4 [-1.4-2.3]	-0.2 [-2.9-2.4]	0.3 [-2.5-3.1]	1.6 [-0.7-4.0]	0.3 [-2.4-3.0]	-0.2 [-2.1-1.6]	-0.5 [-2.9-1.8]	0.4 [-1.6-2.4]	-2.4 [-5.0-0.1]	0.8 [-1.1-2.8]	0.2 [-2.0-2.6]	0.2 [-2.0-2.4]	1.4 [-1.1-3.9]	0.8 [-1.5-3.1]	-0.5 [-2.8-1.7]	1.2 [-1.6-4.0]	0.7 [-1.6-3.0]	0.6 [-2.2-3.4]	1.4 [-1.0-3.7]	1.5 [-0.9-3.8]	-0.0 [-2.4-2.2]	0.8 [-1.8-3.4]	0.8 [-1.5-3.0]	0.8 [-1.8-3.6]	0.9 [-1.9-3.6]	1.1 [-1.5-3.5]	1.3 [-1.4-4.0]	1.4 [-1.3-4.1]	1.5 [-0.9-3.9]	1.3 [-0.8-3.4]	-0.4 [-2.5-1.7]	1.8 [-0.9-4.5]	0.6 [-2.1-3.3]	0.8 [-1.9-3.5]	1.5 [-1.4-4.4]	1.2 [-1.1-3.5]	0.3 [-2.1-2.8]	1.5 [-1.1-4.3]	1.5 [-0.6-3.5]	1.7 [-1.0-4.4]	1.3 [-1.5-4.0]	1.4 [-1.0-3.9]	1.9 [-0.7-4.6]	1.3 [-1.4-4.1]	-0.3 [-3.2-2.3]
CITp				-0.2 [-2.8-2.4]	0.3 [-2.1-2.6]	0.1 [-2.0-2.2]	-0.4 [-2.5-1.7]	-1.1 [-4.0-1.7]	-0.6 [-3.5-2.2]	0.7 [-1.8-3.2]	-0.6 [-3.4-2.2]	-1.1 [-3.1-0.8]	-1.4 [-3.8-1.1]	-0.5 [-2.7-1.7]	-3.3 [-6.0–0.7]	-0.1 [-2.2-2.1]	-0.6 [-3.1-1.7]	-0.6 [-3.0-1.7]	0.5 [-2.1-3.1]	-0.1 [-2.4-2.3]	-1.4 [-3.8-1.0]	0.3 [-2.6-3.4]	-0.2 [-2.6-2.2]	-0.2 [-3.2-2.6]	0.5 [-2.1-3.0]	0.6 [-2.0-3.2]	-0.9 [-3.5-1.6]	-0.0 [-2.9-2.8]	-0.1 [-2.3-2.2]	-0.1 [-2.7-2.6]	0.0 [-2.8-2.8]	0.2 [-2.3-2.6]	0.4 [-2.4-3.2]	0.5 [-2.3-3.4]	0.6 [-1.9-3.3]	0.5 [-1.8-2.7]	-1.3 [-3.6-1.0]	0.9 [-1.8-3.8]	-0.3 [-3.2-2.5]	-0.1 [-2.9-2.8]	0.7 [-2.3-3.6]	0.3 [-2.1-2.8]	-0.5 [-3.2-2.0]	0.7 [-2.1-3.5]	0.6 [-1.7-2.8]	0.8 [-2.0-3.7]	0.4 [-2.5-3.3]	0.6 [-2.0-3.2]	1.1 [-1.7-3.9]	0.4 [-2.4-3.3]	-1.2 [-4.1-1.6]
DESp					0.5 [-2.0-3.0]	0.3 [-2.1-2.8]	-0.2 [-2.5-2.0]	-0.9 [-4.0-2.1]	-0.4 [-3.4-2.6]	0.9 [-1.7-3.6]	-0.4 [-3.3-2.5]	-0.9 [-3.2-1.3]	-1.2 [-3.9-1.5]	-0.3 [-2.7-2.2]	-3.1 [-6.0–0.3]	0.1 [-2.2-2.5]	-0.4 [-3.1-2.2]	-0.4 [-2.9-2.0]	0.7 [-2.1-3.5]	0.1 [-2.5-2.8]	-1.2 [-3.8-1.3]	0.5 [-2.6-3.6]	-0.0 [-2.7-2.7]	-0.0 [-3.1-3.0]	0.7 [-2.1-3.4]	0.8 [-2.0-3.6]	-0.7 [-3.5-2.0]	0.2 [-2.9-3.1]	0.1 [-2.6-2.7]	0.1 [-2.8-3.0]	0.2 [-2.8-3.2]	0.4 [-2.4-3.1]	0.6 [-2.1-3.3]	0.8 [-2.2-3.8]	0.8 [-1.9-3.6]	0.7 [-1.7-3.1]	-1.1 [-3.5-1.3]	1.1 [-1.8-4.1]	-0.1 [-3.2-2.9]	0.1 [-2.8-3.1]	0.9 [-2.2-3.9]	0.5 [-2.1-3.2]	-0.3 [-3.1-2.4]	0.9 [-2.1-3.9]	0.8 [-1.7-3.3]	1.0 [-2.0-4.1]	0.6 [-2.4-3.6]	0.8 [-2.0-3.6]	1.3 [-1.7-4.2]	0.6 [-2.4-3.7]	-1.0 [-4.1-1.9]
DULp						-0.1 [-2.4-2.1]	-0.7 [-2.8-1.4]	-1.4 [-4.3-1.5]	-0.9 [-3.8-2.0]	0.5 [-2.0-3.0]	-0.9 [-3.6-1.9]	-1.4 [-3.3-0.6]	-1.7 [-4.2-0.8]	-0.7 [-3.1-1.6]	-3.6 [-6.4–0.9]	-0.4 [-2.5-1.8]	-0.9 [-3.3-1.6]	-0.9 [-3.1-1.4]	0.2 [-2.4-2.8]	-0.3 [-2.8-2.1]	-1.7 [-4.2-0.8]	0.1 [-2.9-3.0]	-0.5 [-3.0-2.0]	-0.5 [-3.4-2.3]	0.2 [-2.4-2.9]	0.3 [-2.2-2.9]	-1.2 [-3.9-1.4]	-0.3 [-3.2-2.6]	-0.4 [-2.8-2.1]	-0.4 [-3.2-2.5]	-0.3 [-3.2-2.6]	-0.1 [-2.7-2.5]	0.1 [-2.7-3.0]	0.3 [-2.6-3.1]	0.4 [-2.2-2.9]	0.2 [-1.8-2.2]	-1.5 [-3.6-0.6]	0.7 [-2.2-3.6]	-0.6 [-3.5-2.3]	-0.3 [-3.2-2.5]	0.4 [-2.5-3.4]	0.1 [-2.4-2.5]	-0.8 [-3.4-1.8]	0.4 [-2.5-3.3]	0.3 [-2.0-2.6]	0.6 [-2.3-3.4]	0.2 [-2.7-3.0]	0.3 [-2.3-2.9]	0.8 [-1.9-3.6]	0.1 [-2.7-3.0]	-1.5 [-4.4-1.3]
ESCp							-0.5 [-2.4-1.3]	-1.2 [-4.0-1.5]	-0.7 [-3.5-2.0]	0.6 [-1.7-3.0]	-0.7 [-3.4-1.9]	-1.2 [-3.0-0.6]	-1.5 [-3.9-0.7]	-0.6 [-2.7-1.4]	-3.4 [-6.0–0.9]	-0.2 [-2.2-1.8]	-0.7 [-3.1-1.6]	-0.8 [-2.9-1.4]	0.4 [-2.1-2.8]	-0.2 [-2.5-2.2]	-1.5 [-3.8-0.7]	0.2 [-2.6-3.0]	-0.3 [-2.6-2.0]	-0.4 [-3.2-2.3]	0.4 [-2.0-2.7]	0.5 [-1.9-2.8]	-1.0 [-3.5-1.5]	-0.2 [-2.9-2.6]	-0.2 [-2.3-1.9]	-0.2 [-2.8-2.5]	-0.1 [-2.9-2.6]	0.1 [-2.3-2.5]	0.3 [-2.5-3.0]	0.4 [-2.2-3.0]	0.5 [-2.0-3.0]	0.3 [-1.8-2.4]	-1.4 [-3.5-0.7]	0.8 [-1.8-3.5]	-0.4 [-3.0-2.2]	-0.2 [-2.9-2.4]	0.5 [-2.3-3.4]	0.2 [-2.1-2.5]	-0.7 [-3.1-1.7]	0.6 [-2.1-3.2]	0.5 [-1.7-2.6]	0.7 [-1.9-3.5]	0.3 [-2.5-3.1]	0.5 [-2.0-2.9]	0.9 [-1.7-3.6]	0.3 [-2.4-3.1]	-1.3 [-4.1-1.3]
FLOp								-0.7 [-3.3-1.8]	-0.2 [-2.8-2.4]	1.2 [-1.0-3.3]	-0.2 [-2.7-2.4]	-0.7 [-2.2-0.8]	-1.0 [-3.1-1.1]	-0.0 [-1.9-1.9]	-2.9 [-5.3–0.5]	0.3 [-1.4-2.1]	-0.2 [-2.4-2.0]	-0.2 [-2.2-1.7]	0.9 [-1.3-3.1]	0.4 [-1.7-2.4]	-1.0 [-3.1-1.1]	0.8 [-1.9-3.5]	0.2 [-2.0-2.3]	0.2 [-2.4-2.8]	0.9 [-1.4-3.2]	1.0 [-1.1-3.3]	-0.5 [-2.8-1.7]	0.4 [-2.2-3.0]	0.3 [-1.8-2.4]	0.3 [-2.2-2.9]	0.4 [-2.2-3.1]	0.6 [-1.6-2.9]	0.8 [-1.7-3.4]	1.0 [-1.6-3.6]	1.1 [-1.1-3.3]	0.9 [-1.0-2.8]	-0.8 [-2.8-1.0]	1.4 [-1.2-3.9]	0.1 [-2.4-2.7]	0.4 [-2.3-3.0]	1.1 [-1.6-3.7]	0.8 [-1.3-2.8]	-0.1 [-2.4-2.1]	1.1 [-1.5-3.7]	1.0 [-0.7-2.8]	1.3 [-1.3-3.8]	0.9 [-1.8-3.4]	1.0 [-1.3-3.3]	1.5 [-1.0-4.0]	0.8 [-1.7-3.5]	-0.8 [-3.4-1.8]
FLVp									0.5 [-2.7-3.8]	1.8 [-1.1-4.8]	0.5 [-2.7-3.7]	0.0 [-2.5-2.6]	-0.3 [-3.3-2.6]	0.6 [-2.1-3.4]	-2.2 [-5.3-0.8]	1.0 [-1.7-3.7]	0.5 [-2.4-3.5]	0.5 [-2.3-3.3]	1.6 [-1.4-4.7]	1.1 [-1.9-3.9]	-0.3 [-3.1-2.5]	1.4 [-1.8-4.8]	0.9 [-1.9-3.8]	0.9 [-2.3-4.1]	1.6 [-1.4-4.6]	1.7 [-1.3-4.7]	0.2 [-2.8-3.2]	1.1 [-2.1-4.3]	1.0 [-1.9-3.9]	1.0 [-2.1-4.3]	1.1 [-2.1-4.4]	1.3 [-1.7-4.3]	1.5 [-1.7-4.7]	1.7 [-1.5-4.9]	1.8 [-1.3-4.7]	1.6 [-1.2-4.3]	-0.2 [-2.9-2.6]	2.1 [-1.1-5.4]	0.8 [-2.5-4.1]	1.0 [-2.2-4.3]	1.8 [-1.6-5.1]	1.4 [-1.5-4.3]	0.6 [-2.5-3.6]	1.8 [-1.5-5.0]	1.7 [-1.0-4.5]	1.9 [-1.3-5.2]	1.5 [-1.7-4.8]	1.7 [-1.3-4.8]	2.2 [-1.0-5.4]	1.5 [-1.6-4.9]	-0.1 [-3.4-3.0]
LEVp										1.3 [-1.5-4.3]	0.0 [-3.1-3.2]	-0.5 [-3.0-2.1]	-0.8 [-3.7-2.2]	0.1 [-2.6-2.9]	-2.7 [-5.8-0.4]	0.5 [-2.1-3.2]	-0.0 [-2.9-2.9]	-0.0 [-2.8-2.8]	1.1 [-1.8-4.2]	0.5 [-2.4-3.5]	-0.8 [-3.7-2.1]	0.9 [-2.4-4.3]	0.4 [-2.6-3.3]	0.4 [-2.9-3.6]	1.1 [-2.0-4.2]	1.2 [-1.8-4.2]	-0.3 [-3.5-2.7]	0.5 [-2.7-3.8]	0.5 [-2.3-3.5]	0.5 [-2.7-3.7]	0.6 [-2.7-3.9]	0.8 [-2.2-3.9]	1.0 [-2.2-4.3]	1.1 [-2.1-4.4]	1.2 [-1.7-4.4]	1.1 [-1.6-3.8]	-0.7 [-3.4-2.1]	1.5 [-1.7-4.8]	0.3 [-2.9-3.5]	0.5 [-2.7-3.9]	1.3 [-2.1-4.5]	0.9 [-2.0-4.0]	0.1 [-2.9-3.1]	1.3 [-2.0-4.5]	1.2 [-1.6-4.0]	1.4 [-1.8-4.7]	1.0 [-2.2-4.3]	1.2 [-1.9-4.2]	1.7 [-1.6-5.0]	1.0 [-2.2-4.3]	-0.6 [-4.0-2.6]
MIRp											-1.3 [-4.1-1.6]	-1.8 [-4.0-0.3]	-2.1 [-4.8-0.5]	-1.2 [-3.6-1.1]	-4.1 [-6.8–1.3]	-0.8 [-3.2-1.5]	-1.4 [-4.0-1.3]	-1.4 [-3.8-1.0]	-0.2 [-3.0-2.5]	-0.8 [-3.4-1.8]	-2.1 [-4.3-0.0]	-0.4 [-3.5-2.5]	-1.0 [-3.5-1.5]	-1.0 [-3.9-1.9]	-0.3 [-3.0-2.4]	-0.1 [-2.8-2.6]	-1.7 [-4.5-1.0]	-0.8 [-3.8-2.1]	-0.8 [-3.3-1.7]	-0.8 [-3.7-2.0]	-0.7 [-3.7-2.2]	-0.6 [-3.2-2.2]	-0.3 [-3.3-2.6]	-0.2 [-3.2-2.7]	-0.1 [-2.8-2.6]	-0.3 [-2.7-2.0]	-2.0 [-4.4-0.3]	0.2 [-2.8-3.1]	-1.1 [-4.0-1.9]	-0.8 [-3.7-2.2]	-0.1 [-3.3-2.9]	-0.4 [-3.0-2.2]	-1.3 [-4.0-1.4]	-0.1 [-3.0-2.9]	-0.2 [-2.6-2.2]	0.1 [-2.9-3.1]	-0.3 [-3.2-2.5]	-0.2 [-2.9-2.5]	0.3 [-2.5-3.2]	-0.3 [-3.3-2.7]	-2.0 [-5.0-1.0]
NEFp												-0.5 [-3.1-2.1]	-0.8 [-3.8-2.1]	0.1 [-2.6-2.7]	-2.7 [-5.8-0.3]	0.5 [-2.1-3.1]	-0.0 [-2.9-2.8]	-0.0 [-2.7-2.7]	1.1 [-1.9-4.1]	0.5 [-2.4-3.4]	-0.8 [-3.7-1.9]	0.9 [-2.3-4.3]	0.4 [-2.4-3.3]	0.4 [-2.9-3.5]	1.1 [-1.9-4.1]	1.2 [-1.8-4.1]	-0.3 [-3.4-2.6]	0.5 [-2.6-3.9]	0.5 [-2.3-3.3]	0.5 [-2.7-3.7]	0.6 [-2.6-3.8]	0.8 [-2.3-3.8]	1.0 [-2.2-4.2]	1.1 [-2.1-4.3]	1.2 [-1.7-4.2]	1.0 [-1.6-3.7]	-0.7 [-3.4-2.0]	1.5 [-1.6-4.7]	0.3 [-3.0-3.5]	0.5 [-2.7-3.7]	1.3 [-2.0-4.6]	0.9 [-2.0-3.8]	0.1 [-3.0-3.1]	1.3 [-1.9-4.4]	1.2 [-1.5-3.9]	1.4 [-1.8-4.7]	1.0 [-2.2-4.3]	1.2 [-1.8-4.2]	1.7 [-1.4-4.8]	1.0 [-2.2-4.2]	-0.6 [-3.9-2.6]
PARp													-0.3 [-2.4-1.8]	0.6 [-1.3-2.5]	-2.2 [-4.6-0.1]	1.0 [-0.7-2.7]	0.5 [-1.6-2.6]	0.5 [-1.4-2.4]	1.6 [-0.7-3.9]	1.0 [-0.9-3.0]	-0.3 [-2.4-1.8]	1.4 [-1.2-4.1]	0.9 [-1.3-3.0]	0.9 [-1.7-3.5]	1.6 [-0.7-3.9]	1.7 [-0.5-4.0]	0.2 [-2.1-2.4]	1.1 [-1.4-3.7]	1.0 [-1.1-3.0]	1.0 [-1.4-3.5]	1.1 [-1.5-3.7]	1.3 [-0.9-3.6]	1.5 [-1.0-4.1]	1.7 [-0.9-4.2]	1.7 [-0.5-4.0]	1.6 [-0.1-3.2]	-0.2 [-1.9-1.6]	2.0 [-0.5-4.6]	0.8 [-1.7-3.3]	1.0 [-1.6-3.6]	1.8 [-0.9-4.4]	1.4 [-0.6-3.3]	0.6 [-1.7-2.8]	1.8 [-0.8-4.3]	1.7 [-0.2-3.6]	1.9 [-0.6-4.5]	1.5 [-1.1-4.1]	1.7 [-0.5-3.9]	2.2 [-0.3-4.7]	1.5 [-1.1-4.1]	-0.1 [-2.8-2.4]
REBp														0.9 [-1.4-3.3]	-1.9 [-4.7-0.8]	1.3 [-0.9-3.6]	0.8 [-1.9-3.5]	0.8 [-1.6-3.2]	1.9 [-0.8-4.6]	1.3 [-1.2-3.9]	-0.0 [-2.5-2.6]	1.7 [-1.3-4.7]	1.2 [-1.3-3.8]	1.2 [-1.8-4.1]	1.9 [-0.8-4.7]	2.0 [-0.6-4.7]	0.5 [-2.2-3.2]	1.4 [-1.6-4.3]	1.3 [-1.2-3.9]	1.3 [-1.5-4.2]	1.4 [-1.6-4.4]	1.6 [-1.1-4.3]	1.8 [-1.1-4.8]	2.0 [-1.0-5.0]	2.1 [-0.6-4.8]	1.9 [-0.4-4.2]	0.1 [-2.2-2.5]	2.3 [-0.5-5.4]	1.1 [-1.8-4.1]	1.3 [-1.6-4.3]	2.1 [-1.0-5.2]	1.7 [-0.8-4.3]	0.9 [-1.6-3.3]	2.1 [-0.8-5.1]	2.0 [-0.4-4.4]	2.2 [-0.8-5.2]	1.8 [-1.1-4.7]	2.0 [-0.5-4.5]	2.5 [-0.3-5.4]	1.8 [-1.1-4.8]	0.2 [-2.8-3.1]
SERp															-2.8 [-5.4–0.4]	0.4 [-1.6-2.5]	-0.2 [-2.5-2.2]	-0.2 [-2.3-2.0]	1.0 [-1.5-3.6]	0.4 [-1.9-2.8]	-0.9 [-3.2-1.3]	0.8 [-2.0-3.7]	0.3 [-1.9-2.4]	0.2 [-2.5-3.0]	0.9 [-1.6-3.5]	1.1 [-1.4-3.5]	-0.4 [-2.8-1.9]	0.4 [-2.3-3.3]	0.4 [-1.9-2.7]	0.4 [-2.3-3.1]	0.5 [-2.3-3.2]	0.7 [-1.8-3.1]	0.9 [-1.9-3.7]	1.0 [-1.8-3.8]	1.1 [-1.3-3.7]	0.9 [-1.2-3.0]	-0.8 [-3.0-1.4]	1.4 [-1.3-4.1]	0.1 [-2.6-2.9]	0.4 [-2.3-3.1]	1.1 [-1.8-4.0]	0.8 [-1.6-3.1]	-0.1 [-2.5-2.5]	1.1 [-1.6-3.8]	1.1 [-1.1-3.3]	1.3 [-1.4-4.1]	0.9 [-1.8-3.6]	1.0 [-1.5-3.6]	1.5 [-1.2-4.3]	0.9 [-1.9-3.8]	-0.7 [-3.6-1.9]
TRAp																3.2 [0.8-5.7]	2.7 [-0.0-5.5]	2.7 [0.1-5.3]	3.8 [0.9-6.8]	3.3 [0.5-6.1]	1.9 [-0.7-4.5]	3.7 [0.5-6.8]	3.1 [0.4-5.8]	3.1 [0.1-6.1]	3.8 [0.9-6.7]	3.9 [1.1-6.7]	2.4 [-0.4-5.3]	3.3 [0.2-6.4]	3.2 [0.5-6.0]	3.2 [0.3-6.4]	3.3 [0.2-6.5]	3.5 [0.7-6.3]	3.7 [0.7-6.9]	3.9 [0.8-7.0]	4.0 [1.1-6.8]	3.8 [1.3-6.4]	2.0 [-0.5-4.6]	4.3 [1.2-7.4]	3.0 [-0.1-6.1]	3.2 [0.2-6.4]	4.0 [0.8-7.2]	3.6 [0.9-6.4]	2.8 [-0.1-5.6]	4.0 [0.9-7.3]	3.9 [1.3-6.5]	4.1 [1.1-7.3]	3.7 [0.7-6.8]	3.9 [1.1-6.9]	4.4 [1.3-7.5]	3.7 [0.7-6.7]	2.1 [-0.9-5.2]
VENp																	-0.5 [-2.8-1.7]	-0.5 [-2.6-1.5]	0.6 [-1.8-3.0]	0.0 [-2.2-2.3]	-1.3 [-3.6-0.9]	0.4 [-2.4-3.2]	-0.1 [-2.4-2.2]	-0.2 [-2.8-2.6]	0.6 [-1.8-2.9]	0.7 [-1.7-3.0]	-0.8 [-3.3-1.6]	0.0 [-2.5-2.7]	0.0 [-2.2-2.2]	-0.0 [-2.6-2.6]	0.1 [-2.6-2.8]	0.3 [-2.2-2.7]	0.5 [-2.2-3.2]	0.6 [-2.1-3.4]	0.7 [-1.6-3.1]	0.5 [-1.5-2.5]	-1.2 [-3.2-0.8]	1.0 [-1.6-3.7]	-0.2 [-2.9-2.5]	0.0 [-2.7-2.8]	0.8 [-2.0-3.5]	0.4 [-1.8-2.6]	-0.4 [-2.9-2.0]	0.8 [-1.9-3.4]	0.7 [-1.2-2.5]	0.9 [-1.8-3.6]	0.5 [-2.2-3.2]	0.7 [-1.8-3.1]	1.2 [-1.4-3.7]	0.5 [-2.1-3.1]	-1.1 [-3.9-1.4]
VILp																		-0.0 [-2.5-2.4]	1.1 [-1.5-3.8]	0.6 [-1.9-3.1]	-0.8 [-3.3-1.7]	1.0 [-2.0-4.0]	0.4 [-2.2-3.1]	0.4 [-2.6-3.3]	1.1 [-1.6-3.8]	1.2 [-1.4-3.9]	-0.3 [-3.0-2.4]	0.6 [-2.3-3.5]	0.5 [-2.0-3.1]	0.5 [-2.3-3.4]	0.6 [-2.3-3.6]	0.8 [-1.6-3.2]	1.0 [-2.0-4.0]	1.2 [-1.8-4.2]	1.3 [-1.4-4.0]	1.1 [-1.3-3.4]	-0.6 [-3.0-1.7]	1.6 [-1.4-4.5]	0.3 [-2.6-3.3]	0.6 [-2.4-3.5]	1.3 [-1.8-4.4]	1.0 [-1.6-3.5]	0.1 [-2.6-2.8]	1.3 [-1.7-4.2]	1.2 [-1.2-3.6]	1.5 [-1.4-4.3]	1.1 [-1.9-4.0]	1.2 [-1.5-3.9]	1.7 [-1.1-4.6]	1.0 [-1.9-4.1]	-0.6 [-3.6-2.4]
VORp																			1.1 [-1.4-3.7]	0.6 [-1.8-2.9]	-0.8 [-3.1-1.7]	1.0 [-1.9-3.9]	0.4 [-2.0-2.8]	0.4 [-2.4-3.2]	1.1 [-1.5-3.7]	1.2 [-1.3-3.8]	-0.3 [-2.8-2.3]	0.6 [-2.2-3.4]	0.5 [-1.8-2.9]	0.5 [-2.1-3.3]	0.6 [-2.2-3.5]	0.8 [-1.7-3.4]	1.0 [-1.7-3.8]	1.2 [-1.7-4.0]	1.3 [-1.2-3.8]	1.1 [-1.0-3.2]	-0.6 [-2.5-1.2]	1.6 [-1.3-4.4]	0.3 [-2.5-3.1]	0.6 [-2.3-3.4]	1.3 [-1.6-4.2]	1.0 [-1.4-3.3]	0.1 [-2.3-2.6]	1.3 [-1.5-4.1]	1.2 [-1.0-3.5]	1.5 [-1.3-4.3]	1.1 [-1.8-3.8]	1.2 [-1.3-3.8]	1.7 [-1.1-4.5]	1.0 [-1.7-3.9]	-0.6 [-3.3-2.0]
AGOFLOp																				-0.6 [-3.2-2.1]	-1.9 [-4.6-0.8]	-0.2 [-3.4-3.0]	-0.7 [-3.5-2.0]	-0.8 [-3.8-2.3]	-0.0 [-2.9-2.9]	0.1 [-2.6-2.9]	-1.4 [-4.3-1.4]	-0.6 [-3.6-2.5]	-0.6 [-3.3-2.0]	-0.6 [-3.6-2.4]	-0.5 [-3.7-2.6]	-0.3 [-3.2-2.5]	-0.1 [-3.1-2.9]	0.0 [-3.1-3.1]	0.1 [-2.7-2.9]	-0.1 [-2.5-2.4]	-1.8 [-4.3-0.8]	0.4 [-2.6-3.5]	-0.8 [-3.9-2.2]	-0.6 [-3.7-2.6]	0.1 [-3.1-3.2]	-0.2 [-2.9-2.5]	-1.0 [-3.8-1.8]	0.2 [-2.9-3.2]	0.1 [-2.4-2.6]	0.3 [-2.8-3.4]	-0.1 [-3.2-3.0]	0.1 [-2.8-2.9]	0.6 [-2.4-3.5]	-0.1 [-3.1-2.9]	-1.7 [-4.9-1.3]
AGOPARp																					-1.3 [-3.9-1.1]	0.4 [-2.6-3.5]	-0.2 [-2.7-2.4]	-0.2 [-3.2-2.7]	0.5 [-2.2-3.2]	0.7 [-2.0-3.3]	-0.9 [-3.5-1.9]	0.0 [-2.9-3.0]	-0.0 [-2.4-2.4]	-0.0 [-2.8-2.9]	0.1 [-2.8-3.0]	0.2 [-2.4-2.9]	0.5 [-2.5-3.3]	0.6 [-2.3-3.6]	0.7 [-1.9-3.3]	0.5 [-1.8-2.8]	-1.2 [-3.5-1.1]	1.0 [-2.0-3.9]	-0.3 [-3.1-2.6]	-0.0 [-3.0-2.9]	0.7 [-2.3-3.7]	0.4 [-2.1-2.9]	-0.5 [-3.2-2.2]	0.7 [-2.2-3.6]	0.6 [-1.7-3.0]	0.9 [-2.1-3.8]	0.5 [-2.4-3.5]	0.6 [-2.0-3.3]	1.1 [-1.8-4.0]	0.5 [-2.4-3.4]	-1.2 [-4.1-1.7]
AMIMIRp																						1.7 [-1.2-4.6]	1.2 [-1.2-3.6]	1.2 [-1.7-3.9]	1.9 [-0.8-4.6]	2.0 [-0.6-4.7]	0.5 [-2.2-3.1]	1.4 [-1.5-4.3]	1.3 [-1.2-3.7]	1.3 [-1.6-4.2]	1.4 [-1.5-4.4]	1.6 [-1.0-4.3]	1.8 [-1.1-4.8]	2.0 [-1.0-4.9]	2.1 [-0.6-4.8]	1.9 [-0.5-4.2]	0.1 [-2.1-2.4]	2.4 [-0.5-5.2]	1.1 [-1.8-3.9]	1.3 [-1.5-4.2]	2.1 [-0.9-5.1]	1.7 [-0.7-4.2]	0.9 [-1.8-3.4]	2.1 [-0.8-4.9]	2.0 [-0.4-4.3]	2.2 [-0.7-5.2]	1.8 [-1.0-4.7]	2.0 [-0.6-4.7]	2.5 [-0.4-5.3]	1.8 [-1.0-4.8]	0.2 [-2.8-3.1]
AMIPARp																							-0.6 [-3.5-2.4]	-0.6 [-3.9-2.7]	0.1 [-3.0-3.2]	0.3 [-2.9-3.4]	-1.3 [-4.4-1.9]	-0.4 [-3.8-3.0]	-0.4 [-3.4-2.5]	-0.4 [-3.8-2.9]	-0.3 [-3.7-3.1]	-0.2 [-3.2-3.0]	0.1 [-3.2-3.3]	0.2 [-3.2-3.6]	0.3 [-2.8-3.5]	0.1 [-2.7-2.9]	-1.6 [-4.5-1.2]	0.6 [-2.7-3.9]	-0.7 [-4.0-2.7]	-0.4 [-3.8-2.9]	0.3 [-3.0-3.8]	-0.0 [-3.0-3.0]	-0.9 [-3.9-2.1]	0.3 [-2.9-3.6]	0.3 [-2.6-3.1]	0.5 [-2.8-3.8]	0.1 [-3.3-3.5]	0.2 [-2.9-3.4]	0.7 [-2.6-4.1]	0.1 [-3.3-3.4]	-1.6 [-4.9-1.8]
AMISERp																								-0.0 [-2.9-2.8]	0.7 [-2.0-3.4]	0.8 [-1.8-3.5]	-0.7 [-3.4-1.9]	0.2 [-2.8-3.1]	0.1 [-2.4-2.7]	0.1 [-2.8-3.0]	0.2 [-2.7-3.2]	0.4 [-2.3-3.0]	0.6 [-2.3-3.5]	0.8 [-2.2-3.6]	0.9 [-1.8-3.5]	0.7 [-1.7-3.0]	-1.1 [-3.4-1.3]	1.2 [-1.8-4.2]	-0.1 [-3.1-2.9]	0.1 [-2.8-3.1]	0.9 [-2.2-4.0]	0.5 [-2.0-3.1]	-0.3 [-3.0-2.4]	0.9 [-2.0-3.8]	0.8 [-1.6-3.2]	1.0 [-1.9-4.1]	0.6 [-2.3-3.6]	0.8 [-1.9-3.5]	1.3 [-1.6-4.2]	0.6 [-2.4-3.6]	-1.0 [-4.0-1.9]
AMITRAp																									0.7 [-2.4-3.8]	0.8 [-2.2-3.9]	-0.7 [-3.8-2.3]	0.2 [-3.1-3.4]	0.2 [-2.8-3.0]	0.1 [-3.1-3.4]	0.2 [-3.0-3.5]	0.4 [-2.7-3.6]	0.6 [-2.5-3.9]	0.8 [-2.5-4.1]	0.9 [-2.1-3.9]	0.7 [-2.0-3.5]	-1.0 [-3.8-1.8]	1.2 [-2.1-4.5]	-0.1 [-3.4-3.1]	0.2 [-3.1-3.5]	0.9 [-2.5-4.2]	0.6 [-2.4-3.5]	-0.3 [-3.3-2.7]	0.9 [-2.3-4.1]	0.8 [-2.0-3.6]	1.1 [-2.3-4.4]	0.7 [-2.7-3.9]	0.8 [-2.3-3.9]	1.3 [-1.9-4.5]	0.7 [-2.6-3.9]	-1.0 [-4.3-2.2]
BUPESCp																										0.1 [-2.6-3.0]	-1.4 [-4.2-1.4]	-0.5 [-3.5-2.6]	-0.6 [-3.1-2.0]	-0.6 [-3.5-2.4]	-0.5 [-3.5-2.5]	-0.3 [-3.1-2.5]	-0.1 [-3.2-3.0]	0.1 [-2.9-3.1]	0.2 [-2.7-3.0]	-0.0 [-2.5-2.5]	-1.7 [-4.3-0.7]	0.5 [-2.6-3.6]	-0.8 [-3.9-2.2]	-0.5 [-3.7-2.4]	0.2 [-3.0-3.4]	-0.1 [-2.9-2.6]	-1.0 [-3.9-1.9]	0.2 [-2.9-3.2]	0.1 [-2.4-2.7]	0.3 [-2.8-3.4]	-0.0 [-3.1-3.0]	0.1 [-2.7-3.0]	0.6 [-2.3-3.6]	-0.1 [-3.1-3.1]	-1.7 [-4.9-1.4]
BUPFLOp																											-1.5 [-4.3-1.3]	-0.6 [-3.7-2.3]	-0.7 [-3.4-1.9]	-0.7 [-3.6-2.3]	-0.6 [-3.7-2.5]	-0.4 [-3.2-2.4]	-0.2 [-3.2-2.8]	-0.0 [-3.1-3.0]	0.1 [-2.7-2.8]	-0.1 [-2.6-2.3]	-1.9 [-4.4-0.6]	0.4 [-2.6-3.4]	-0.9 [-3.9-2.1]	-0.7 [-3.7-2.4]	0.1 [-3.1-3.2]	-0.3 [-2.9-2.4]	-1.1 [-3.9-1.6]	0.1 [-2.9-3.1]	-0.0 [-2.4-2.4]	0.2 [-2.8-3.2]	-0.2 [-3.3-2.9]	-0.0 [-2.9-2.8]	0.5 [-2.5-3.5]	-0.2 [-3.2-2.8]	-1.8 [-4.9-1.2]
BUPSERp																												0.9 [-2.1-3.9]	0.8 [-1.8-3.5]	0.8 [-2.1-3.9]	0.9 [-2.1-4.0]	1.1 [-1.8-3.9]	1.3 [-1.7-4.5]	1.5 [-1.6-4.5]	1.6 [-1.3-4.4]	1.4 [-1.1-4.0]	-0.4 [-2.9-2.2]	1.9 [-1.1-5.0]	0.6 [-2.5-3.6]	0.8 [-2.2-3.9]	1.6 [-1.5-4.9]	1.3 [-1.4-3.9]	0.4 [-2.4-3.2]	1.6 [-1.4-4.6]	1.5 [-1.0-4.1]	1.7 [-1.3-4.9]	1.3 [-1.7-4.4]	1.5 [-1.4-4.3]	2.0 [-1.0-5.0]	1.3 [-1.7-4.5]	-0.3 [-3.4-2.7]
BUPVENp																													-0.0 [-3.0-2.8]	-0.0 [-3.2-3.2]	0.1 [-3.2-3.4]	0.2 [-2.9-3.2]	0.5 [-2.8-3.7]	0.6 [-2.7-3.8]	0.7 [-2.3-3.7]	0.5 [-2.2-3.2]	-1.2 [-4.0-1.4]	1.0 [-2.2-4.2]	-0.3 [-3.6-2.9]	-0.0 [-3.3-3.2]	0.7 [-2.7-4.0]	0.4 [-2.6-3.2]	-0.5 [-3.6-2.6]	0.7 [-2.6-4.0]	0.6 [-2.2-3.3]	0.9 [-2.3-4.1]	0.5 [-2.9-3.8]	0.6 [-2.4-3.7]	1.1 [-2.1-4.4]	0.5 [-2.8-3.7]	-1.2 [-4.4-2.1]
CITESCp																														-0.0 [-2.8-2.8]	0.1 [-2.9-3.0]	0.3 [-2.3-2.9]	0.5 [-2.4-3.4]	0.6 [-2.3-3.5]	0.7 [-1.9-3.4]	0.5 [-1.8-2.8]	-1.2 [-3.5-1.1]	1.0 [-1.8-3.8]	-0.2 [-3.0-2.6]	0.0 [-2.8-2.8]	0.8 [-2.3-3.8]	0.4 [-2.1-2.9]	-0.4 [-3.2-2.2]	0.8 [-2.1-3.6]	0.7 [-1.7-3.0]	0.9 [-2.0-3.9]	0.5 [-2.5-3.4]	0.7 [-2.1-3.4]	1.2 [-1.6-4.0]	0.5 [-2.4-3.5]	-1.1 [-4.1-1.7]
CITPARp																															0.1 [-3.1-3.4]	0.3 [-2.8-3.2]	0.5 [-2.7-3.6]	0.6 [-2.6-3.8]	0.7 [-2.3-3.6]	0.5 [-2.1-3.1]	-1.2 [-3.9-1.5]	1.0 [-2.1-4.2]	-0.2 [-3.5-3.0]	0.0 [-3.3-3.2]	0.8 [-2.7-4.0]	0.4 [-2.4-3.3]	-0.4 [-3.4-2.6]	0.8 [-2.4-3.9]	0.7 [-2.1-3.4]	0.9 [-2.4-4.1]	0.5 [-2.8-3.7]	0.7 [-2.3-3.7]	1.2 [-2.0-4.3]	0.5 [-2.7-3.7]	-1.1 [-4.4-2.1]
CITSERp																																0.2 [-2.9-3.2]	0.4 [-2.9-3.7]	0.5 [-2.7-3.9]	0.6 [-2.4-3.7]	0.5 [-2.3-3.2]	-1.3 [-4.1-1.5]	0.9 [-2.4-4.2]	-0.3 [-3.6-2.9]	-0.1 [-3.3-3.3]	0.7 [-2.7-4.0]	0.3 [-2.6-3.3]	-0.5 [-3.7-2.5]	0.7 [-2.6-3.9]	0.6 [-2.2-3.4]	0.8 [-2.5-4.2]	0.4 [-2.9-3.8]	0.6 [-2.5-3.7]	1.1 [-2.2-4.4]	0.4 [-2.9-3.7]	-1.2 [-4.5-2.0]
CITVILp																																	0.2 [-2.8-3.3]	0.4 [-2.7-3.5]	0.5 [-2.4-3.3]	0.3 [-2.2-2.8]	-1.5 [-4.0-1.1]	0.8 [-2.3-3.8]	-0.5 [-3.6-2.5]	-0.3 [-3.3-2.8]	0.5 [-2.6-3.6]	0.2 [-2.5-2.8]	-0.7 [-3.5-2.1]	0.5 [-2.6-3.5]	0.4 [-2.1-3.0]	0.6 [-2.3-3.7]	0.2 [-2.8-3.3]	0.4 [-2.4-3.2]	0.9 [-2.1-3.9]	0.2 [-2.9-3.3]	-1.4 [-4.5-1.6]
DESDULp																																		0.1 [-3.1-3.4]	0.2 [-2.8-3.3]	0.0 [-2.6-2.8]	-1.7 [-4.4-1.0]	0.5 [-2.7-3.8]	-0.7 [-4.0-2.6]	-0.5 [-3.7-2.8]	0.3 [-3.1-3.5]	-0.1 [-3.0-2.8]	-0.9 [-3.9-2.1]	0.3 [-2.9-3.5]	0.2 [-2.6-3.0]	0.4 [-2.8-3.7]	0.0 [-3.3-3.3]	0.2 [-2.8-3.2]	0.7 [-2.5-4.0]	0.0 [-3.3-3.4]	-1.6 [-5.0-1.6]
DULESCp																																			0.1 [-2.9-3.1]	-0.1 [-2.9-2.6]	-1.8 [-4.6-0.9]	0.4 [-2.8-3.7]	-0.9 [-4.1-2.5]	-0.6 [-3.9-2.6]	0.1 [-3.2-3.4]	-0.2 [-3.2-2.7]	-1.1 [-4.1-2.0]	0.1 [-3.1-3.3]	0.0 [-2.7-2.9]	0.3 [-2.9-3.6]	-0.1 [-3.5-3.2]	0.0 [-3.0-3.1]	0.5 [-2.7-3.7]	-0.1 [-3.4-3.1]	-1.8 [-5.1-1.5]
DULFLOp																																				-0.2 [-2.7-2.2]	-1.9 [-4.3-0.5]	0.3 [-2.7-3.3]	-1.0 [-4.0-2.0]	-0.7 [-3.8-2.4]	0.0 [-3.0-3.1]	-0.3 [-3.0-2.3]	-1.2 [-4.0-1.6]	0.0 [-3.0-3.0]	-0.1 [-2.5-2.5]	0.2 [-2.8-3.2]	-0.2 [-3.3-2.8]	-0.1 [-2.9-2.7]	0.4 [-2.6-3.4]	-0.2 [-3.2-2.8]	-1.9 [-4.9-1.1]
DULPARp																																					-1.7 [-3.7-0.2]	0.5 [-2.2-3.2]	-0.8 [-3.5-2.0]	-0.5 [-3.3-2.2]	0.2 [-2.6-3.0]	-0.1 [-2.4-2.2]	-1.0 [-3.5-1.5]	0.2 [-2.5-3.0]	0.1 [-2.0-2.3]	0.4 [-2.3-3.1]	-0.0 [-2.9-2.8]	0.1 [-2.3-2.5]	0.6 [-2.1-3.3]	-0.0 [-2.8-2.8]	-1.7 [-4.4-1.0]
DULVORp																																						2.2 [-0.5-5.0]	1.0 [-1.8-3.6]	1.2 [-1.6-4.0]	1.9 [-0.8-4.8]	1.6 [-0.7-3.9]	0.7 [-1.7-3.2]	1.9 [-0.8-4.7]	1.9 [-0.3-4.0]	2.1 [-0.7-4.9]	1.7 [-1.1-4.5]	1.8 [-0.6-4.4]	2.3 [-0.4-5.2]	1.7 [-1.0-4.4]	0.1 [-2.7-2.7]
ESCFLOp																																							-1.3 [-4.5-1.9]	-1.0 [-4.3-2.2]	-0.3 [-3.7-3.1]	-0.6 [-3.6-2.2]	-1.5 [-4.5-1.6]	-0.3 [-3.6-3.0]	-0.4 [-3.2-2.4]	-0.1 [-3.3-3.2]	-0.5 [-3.9-2.7]	-0.4 [-3.4-2.7]	0.1 [-3.1-3.3]	-0.5 [-3.8-2.7]	-2.2 [-5.4-1.0]
ESCPARp																																								0.3 [-3.1-3.5]	1.0 [-2.4-4.3]	0.7 [-2.3-3.6]	-0.2 [-3.3-2.8]	1.0 [-2.3-4.2]	0.9 [-1.8-3.7]	1.1 [-2.2-4.5]	0.8 [-2.5-4.1]	0.9 [-2.1-3.9]	1.4 [-1.7-4.6]	0.7 [-2.6-4.0]	-0.9 [-4.2-2.3]
ESCSERp																																									0.7 [-2.6-4.1]	0.4 [-2.5-3.4]	-0.5 [-3.5-2.6]	0.7 [-2.5-4.0]	0.7 [-2.2-3.5]	0.9 [-2.4-4.1]	0.5 [-2.8-3.8]	0.6 [-2.4-3.7]	1.1 [-2.0-4.4]	0.5 [-2.8-3.7]	-1.1 [-4.4-2.1]
FLOMIRp																																										-0.3 [-3.3-2.6]	-1.2 [-4.4-1.9]	0.0 [-3.3-3.4]	-0.1 [-2.8-2.8]	0.2 [-3.2-3.5]	-0.2 [-3.6-3.2]	-0.1 [-3.3-3.1]	0.4 [-2.9-3.7]	-0.3 [-3.6-3.1]	-1.9 [-5.4-1.4]
FLOPARp																																											-0.9 [-3.5-1.8]	0.3 [-2.5-3.2]	0.3 [-2.0-2.5]	0.5 [-2.4-3.5]	0.1 [-2.9-3.0]	0.2 [-2.4-2.8]	0.7 [-2.1-3.6]	0.1 [-2.8-3.0]	-1.6 [-4.5-1.4]
FLOREBp																																												1.2 [-1.8-4.3]	1.1 [-1.3-3.6]	1.4 [-1.7-4.4]	1.0 [-2.1-4.0]	1.1 [-1.6-3.8]	1.6 [-1.4-4.5]	0.9 [-2.1-4.0]	-0.7 [-3.8-2.3]
FLOSERp																																													-0.1 [-2.9-2.6]	0.2 [-3.1-3.4]	-0.2 [-3.6-3.0]	-0.1 [-3.1-3.0]	0.4 [-2.8-3.6]	-0.3 [-3.5-3.0]	-1.9 [-5.2-1.3]
FLOVENp																																														0.2 [-2.5-3.0]	-0.2 [-3.0-2.6]	-0.0 [-2.5-2.6]	0.5 [-2.3-3.2]	-0.2 [-2.9-2.6]	-1.8 [-4.6-0.9]
FLOVILp																																															-0.4 [-3.7-2.9]	-0.3 [-3.3-2.8]	0.2 [-2.9-3.5]	-0.4 [-3.7-2.9]	-2.0 [-5.4-1.2]
MIRTRAp																																																0.1 [-2.9-3.3]	0.6 [-2.5-3.9]	-0.0 [-3.2-3.3]	-1.6 [-5.0-1.7]
PARREBp																																																	0.5 [-2.5-3.5]	-0.2 [-3.2-2.9]	-1.8 [-4.9-1.3]
PARVENp																																																		-0.7 [-3.8-2.5]	-2.3 [-5.6-0.9]
TRAVENp																																																			-1.6 [-5.0-1.5]
VENVORp																																																				

4 NMA covariate adjusted models

To test the robustness of the primary analysis, adjustment for various trial-level covariates was conducted following previous work.[1,2]

- **Study center:** single- versus multi-center trials.
- **Study dosing:** fixed versus flexible dose trials.
- **Study length:** duration of treatment, range 4 - 12 weeks.
- **Study size:** sample size, number of patients randomized.
- **Study year (continuous):** study year was defined as study year of completion, study year of publication, or year of drug approval from the FDA (US Food and Drug Administration), where available in this order; preference was given to study year of completion, because unpublished trials, by definition, have no year of publication, following previous work.[2] The resulting study year range was 1979 - 2014.
- **Study year (categorical):** before versus after year 2000.
- **Publication status:** published versus unpublished trials.
- **Sponsorship:** sponsored versus unsponsored trials.

Continuous covariates were standardized by centering at the mean and dividing by the standard deviation, because standardization results in effect sizes that are on comparable scales when derived from covariates with different units, as follows:

$$x_i = (\text{value}_i - \text{value of centralization})/\text{standard deviation} \qquad \text{(Eq. S1)}$$

Categorical covariates were coded as follows:

$$\text{Study center}_i = \begin{cases} 1, & \text{if trial i is a multi-center trial} \\ 0, & \text{if trial i is a single-center trial} \end{cases} \tag{Eq. S2}$$

$$\text{Study dosing}_i = \begin{cases} 1, & \text{if trial i is a flexible dose trial} \\ 0, & \text{if trial i is a fixed dose trial} \end{cases} \tag{Eq. S3}$$

$$\text{Study year (categorical)}_i = \begin{cases} 1, & \text{if trial i was conducted before 2000} \\ 0, & \text{if trial i was conducted after 2000} \end{cases} \tag{Eq. S3}$$

$$\text{Publication status}_i = \begin{cases} 1, & \text{if trial i is published} \\ 0, & \text{if trial i is unpublished} \end{cases} \tag{Eq. S4}$$

$$\text{Sponsorship}_i = \begin{cases} 1, & \text{if trial i is sponsored} \\ 0, & \text{if trial i is unsponsored} \end{cases} \tag{Eq. S5}$$

Table S 4a: Covariate statistics. Listed are summary statistics for the various covariates, in terms of between-trial heterogeneity (σ), effective number of parameters (pD), residual deviance ($\bar{D}$), deviance information criterion (DIC), and the standardized common beta estimates (B [credible intervals, 95% CrI]). Significant effects (CrIs excluding zero) are highlighted (blue).

	σ	**pD**	$\bar{D}$	**DIC**	***B* [95% CrI]**
Model unadjusted	4.411	647	667	-1286	
Study center	3.271	578	597	-1197	-3.82 [-8.29 - 1.31]
Study dosing	4.327	643	663	-1294	0.42 [-17.34 - 19.41]
Study length	3.981	642	660	-1275	6.06 [-1.66 - 13.84]
Study size	3.816	647	663	-1289	-6.01 [-11.60 - -1.36]
Study year (continuous)	3.791	646	665	-1287	-4.57 [-9.32 - 0.71]
Study year (categorical)	4.068	647	666	-1287	-12.81 [-65.54 - 30.30]
Publication status	3.809	641	660	-1276	-3.72 [-7.58 - 0.34]
Sponsorship	4.383	625	641	-1263	4.51 [-18.52 - 22.33]

Table S 4b: Covariate estimates. Listed are standardized covariate-adjusted beta estimates [credible intervals, 95% CrI] for each drug and placebo. The first line represent the common beta estimate (B). Significant effects (CrI excluding zero) are highlighted (blue). VENVORp was chosen as reference treatment.

	Study center	Study dosing	Study length	Study size	Study year (continuous)	Study year (categorical)	Publcation status	Sponsorship
B	-3.8[-8.3-1.3]	0.4[-17.3-19.4]	6.1[-1.7-13.8]	-6.0[-11.6–1.4]	-4.6[-9.3-0.7]	-12.8[-65.5-30.3]	-3.7[-7.6-0.3]	4.5[-18.5-22.3]
AGO	-4.1[-9.2-1.4]	0.3[-17.7-19.4]	6.1[-1.5-14.2]	-5.8[-11.5–0.9]	-4.6[-9.5-0.8]	-12.8[-65.5-30.4]	-4.8[-9.2–0.2]	4.4[-18.7-22.2]
AMI	-3.1[-7.9-2.4]	0.4[-17.3-19.5]	6.8[-0.9-14.7]	-5.0[-10.7–0.2]	-3.6[-8.5-1.8]	-14.9[-68.1-28.1]	-5.5[-9.7–1.0]	4.5[-18.6-22.5]
BUP	-3.9[-9.0-1.6]	0.1[-17.6-19.2]	6.6[-1.2-14.5]	-5.3[-11.0–0.5]	-3.9[-8.8-1.5]	-13.6[-66.3-29.5]	-4.6[-8.8–0.3]	4.3[-18.8-22.2]
CIT	-4.6[-9.6-1.1]	0.3[-17.4-19.4]	6.4[-1.4-14.4]	-6.2[-11.8–1.5]	-4.4[-9.4-1.1]	-13.9[-66.5-29.1]	-5.0[-9.3–0.6]	4.3[-18.8-22.0]
DES	-4.5[-9.8-1.2]	0.5[-17.1-19.6]	5.9[-1.8-13.9]	-6.4[-12.0–1.6]	-4.8[-9.8-0.7]	-12.8[-65.6-30.4]	-4.9[-9.3–0.1]	4.3[-18.8-22.2]
DUL	-4.8[-10.0-0.7]	0.4[-17.2-19.5]	5.6[-2.2-13.6]	-6.4[-12.0–1.6]	-4.9[-9.7-0.5]	-13.5[-66.3-29.6]	-5.6[-9.7–1.3]	4.5[-18.6-22.3]
ESC	-4.5[-9.5-1.1]	0.5[-17.1-19.6]	5.9[-1.8-13.9]	-6.5[-12.1–1.7]	-4.5[-9.4-1.0]	-13.7[-66.2-29.4]	-4.8[-9.0–0.2]	4.3[-18.8-22.1]
FLO	-2.8[-7.6-2.7]	0.0[-17.5-19.2]	5.9[-2.0-13.8]	-6.1[-11.8–1.3]	-4.1[-9.0-1.3]	-13.7[-66.4-29.3]	-4.4[-8.4–0.1]	4.3[-18.7-22.1]
FLV	-3.5[-8.7-2.3]	0.2[-17.2-19.4]	6.1[-1.8-14.1]	-5.9[-11.7–0.9]	-4.5[-9.6-0.9]	-13.8[-66.7-29.2]	-5.0[-9.8-0.1]	4.3[-18.8-22.1]
LEV	-4.3[-9.6-1.5]	0.4[-17.2-19.5]	5.7[-2.2-13.7]	-6.4[-12.0–1.4]	-4.8[-9.8-0.8]	-12.8[-65.3-30.3]	-4.6[-9.4-0.5]	4.4[-18.8-22.3]
MIR	-2.6[-7.7-3.1]	-0.1[-17.8-19.1]	6.5[-1.4-14.5]	-5.5[-11.2–0.5]	-4.7[-9.6-0.8]	-14.1[-67.0-28.8]	-6.0[-10.5–1.5]	4.4[-18.7-22.3]
NEF	-4.1[-9.4-1.6]	0.3[-17.2-19.5]	6.1[-1.6-14.2]	-5.7[-11.5–0.7]	-4.5[-9.5-1.0]	-13.5[-66.2-29.4]	-4.5[-9.3-0.4]	4.4[-18.7-22.2]
PAR	-3.4[-8.1-2.0]	0.1[-17.6-19.2]	6.1[-1.7-14.0]	-5.7[-11.4–1.0]	-4.6[-9.5-0.7]	-12.6[-65.3-30.4]	-4.8[-8.8–0.5]	4.2[-18.7-22.0]
REB	-6.1[-11.1–0.7]	0.8[-16.9-19.9]	6.4[-1.4-14.4]	-5.3[-10.9–0.5]	-4.3[-9.4-1.3]	-14.0[-66.7-29.1]	-6.3[-10.7–1.7]	4.3[-18.8-22.2]
SER	-3.7[-8.8-1.9]	0.3[-17.4-19.4]	6.0[-1.6-13.9]	-6.0[-11.6–1.1]	-4.6[-9.5-0.9]	-13.1[-66.0-29.9]	-4.9[-9.2–0.6]	4.4[-18.7-22.3]
TRA	-2.2[-7.3-3.5]	0.1[-17.4-19.3]	7.7[-0.0-15.6]	-4.3[-9.9-0.8]	-3.5[-8.4-2.0]	-14.9[-67.8-28.1]	-5.5[-9.9–0.8]	4.0[-18.9-21.8]
VEN	-5.4[-10.3–0.1]	0.3[-17.2-19.5]	6.4[-1.4-14.3]	-5.5[-11.1–0.8]	-4.0[-8.8-1.4]	-14.3[-67.1-28.6]	-5.2[-9.2–0.9]	4.4[-18.7-22.2]
VIL	-4.7[-9.9-0.9]	0.5[-17.0-19.7]	5.8[-1.9-13.8]	-6.5[-12.2–1.8]	-5.1[-10.1-0.4]	-12.8[-65.4-30.1]	-5.2[-9.8–0.6]	4.4[-18.7-22.2]
VOR	-4.2[-9.3-1.2]	0.4[-17.3-19.5]	6.1[-1.2-13.8]	-6.2[-11.9–1.3]	-4.8[-9.8-0.7]	-12.8[-65.0-30.2]	-4.5[-8.4–0.3]	4.4[-18.7-22.3]
AGOp	-3.1[-8.3-2.6]	0.7[-16.9-19.8]	5.8[-2.0-13.8]	-5.8[-11.5–1.0]	-4.0[-9.0-1.5]	-12.8[-65.6-30.4]	-2.7[-7.1-2.0]	4.7[-18.5-22.7]
AMIp	-4.9[-10.0-0.6]	0.4[-17.3-19.5]	5.1[-2.6-13.0]	-7.6[-13.4–2.6]	-5.5[-10.4–0.0]	-11.0[-63.3-32.1]	-1.5[-6.1-3.0]	4.5[-18.6-22.3]
BUPp	-4.0[-9.1-1.6]	0.8[-16.8-19.8]	5.5[-2.4-13.4]	-6.9[-12.5–2.1]	-5.5[-10.5–0.0]	-11.7[-64.6-31.3]	-3.2[-7.5-1.2]	4.7[-18.5-22.7]
CITp	-3.8[-8.9-1.8]	0.4[-17.0-19.5]	5.8[-2.0-13.9]	-6.1[-11.8–1.1]	-4.5[-9.6-0.9]	-13.0[-65.7-30.0]	-3.7[-8.1-0.9]	4.4[-18.7-22.2]
DESp	-3.7[-8.9-1.9]	0.3[-17.3-19.5]	6.1[-1.6-14.1]	-6.1[-11.8–1.2]	-4.5[-9.4-1.1]	-12.8[-65.6-30.2]	-3.4[-8.0-1.3]	4.6[-18.6-22.5]
DULp	-3.8[-8.9-1.7]	0.4[-17.2-19.5]	6.4[-1.5-14.5]	-5.7[-11.6–0.8]	-4.9[-9.9-0.7]	-12.8[-65.6-30.4]	-3.3[-7.7-1.2]	4.7[-18.5-22.6]
ESCp	-3.8[-8.9-1.7]	0.3[-17.4-19.4]	5.9[-1.8-14.0]	-6.1[-11.7–1.2]	-4.6[-9.6-0.7]	-12.8[-65.2-30.4]	-4.2[-8.5-0.2]	4.7[-18.4-22.6]
FLOp	-5.6[-10.4–0.1]	0.8[-16.6-19.9]	5.8[-2.0-13.7]	-6.9[-12.6–2.2]	-5.2[-10.1-0.3]	-12.2[-64.8-30.8]	-2.8[-7.0-1.5]	4.8[-18.5-22.8]
FLVp	-4.1[-9.3-1.5]	0.6[-17.1-19.7]	6.0[-1.8-14.0]	-6.1[-11.9–1.2]	-4.6[-9.6-0.9]	-11.8[-64.4-31.1]	-2.5[-7.3-2.5]	4.7[-18.5-22.6]
LEVp	-3.3[-8.7-2.6]	0.4[-17.1-19.6]	6.4[-1.4-14.4]	-5.7[-11.3–0.8]	-4.3[-9.3-1.2]	-12.8[-65.3-30.2]	-2.9[-7.7-2.0]	4.7[-18.6-22.5]
MIRp	-5.0[-10.2-0.6]	0.6[-17.0-19.7]	6.2[-1.7-14.3]	-5.6[-11.2–0.8]	-4.1[-9.1-1.4]	-13.7[-66.4-29.2]	-3.3[-7.9-1.3]	4.4[-18.7-22.2]
NEFp	-3.5[-8.6-2.2]	0.5[-17.1-19.7]	5.9[-1.8-14.0]	-6.4[-12.3–1.4]	-4.6[-9.7-0.9]	-12.1[-64.8-30.7]	-2.9[-7.7-2.0]	4.6[-18.5-22.5]
PARp	-4.4[-9.2-1.0]	0.7[-16.8-19.8]	5.9[-1.9-13.7]	-6.5[-12.2–1.8]	-5.1[-9.9-0.3]	-12.0[-64.6-31.0]	-2.7[-7.0-1.6]	4.6[-18.6-22.5]
REBp	0.9[-4.4-6.8]	0.1[-17.5-19.3]	5.8[-2.0-13.6]	-7.5[-13.2–2.4]	-4.5[-9.5-1.1]	-11.6[-64.2-31.4]	2.3[-2.9-7.6]	4.7[-18.5-22.6]
SERp	-4.1[-9.1-1.4]	0.5[-17.0-19.6]	6.2[-1.6-14.1]	-6.4[-12.1–1.5]	-4.5[-9.5-1.0]	-12.6[-65.4-30.4]	-3.1[-7.5-1.4]	4.6[-18.6-22.6]
TRAp	-5.4[-10.8-0.2]	1.0[-16.5-20.1]	4.1[-3.7-12.1]	-8.3[-14.1–3.4]	-6.2[-11.2–0.8]	-7.9[-60.3-35.4]	-0.3[-4.9-4.4]	5.2[-18.3-23.1]
VENp	-3.2[-8.1-2.3]	0.2[-17.4-19.4]	5.3[-2.4-13.2]	-6.4[-12.1–1.6]	-4.8[-9.7-0.6]	-12.7[-65.5-30.1]	-3.0[-7.4-1.3]	4.6[-18.6-22.5]
VILp	-3.3[-8.5-2.4]	0.3[-17.2-19.5]	6.3[-1.6-14.3]	-5.2[-10.9–0.3]	-4.0[-9.0-1.4]	-12.8[-65.4-30.3]	-2.8[-7.4-2.0]	4.6[-18.6-22.5]
VORp	-3.2[-8.3-2.3]	0.4[-17.2-19.6]	6.5[-1.1-14.5]	-5.5[-11.1–0.7]	-4.2[-9.1-1.3]	-12.8[-65.5-30.3]	-2.5[-6.6-1.8]	4.6[-18.5-22.6]
AGOFLOp	-3.9[-9.1-1.6]	0.4[-17.2-19.7]	6.0[-1.8-14.0]	-6.1[-11.9–1.2]	-4.5[-9.6-1.0]	-12.8[-65.5-30.4]	-3.9[-8.5-0.7]	4.5[-18.6-22.4]
AGOPARp	-3.6[-8.7-2.0]	0.4[-17.1-19.6]	6.3[-1.6-14.3]	-5.8[-11.5–0.8]	-4.5[-9.5-1.0]	-12.8[-65.6-30.2]	-3.8[-8.4-0.7]	4.6[-18.6-22.5]
AMIMIRp	-3.8[-9.3-2.0]	0.7[-16.8-19.8]	5.6[-2.2-13.5]	-6.6[-12.3–1.6]	-4.9[-9.9-0.6]	-11.6[-64.3-31.6]	-3.0[-7.6-1.6]	4.5[-18.6-22.4]
AMIPARp	-3.7[-8.9-2.0]	0.4[-17.2-19.6]	6.0[-1.9-14.1]	-5.9[-11.8–0.9]	-4.6[-9.5-1.0]	-12.8[-65.5-30.2]	-3.7[-8.9-1.5]	4.5[-18.6-22.4]
AMISERp	-3.3[-8.5-2.3]	0.5[-17.2-19.6]	6.3[-1.5-14.3]	-5.5[-11.4–0.5]	-4.7[-9.7-0.8]	-12.4[-65.1-30.7]	-3.3[-7.8-1.3]	4.5[-18.6-22.3]
AMITRAp	-3.8[-9.2-2.1]	0.5[-17.1-19.7]	5.9[-1.9-13.8]	-6.2[-12.0–1.1]	-4.6[-9.6-0.9]	-12.4[-65.2-30.8]	-3.1[-7.8-1.8]	4.5[-18.6-22.5]
BUPESCp	-3.9[-9.2-1.9]	0.4[-17.3-19.6]	6.1[-1.7-14.1]	-6.0[-11.7–1.0]	-4.6[-9.6-0.9]	-12.8[-65.6-30.3]	-3.8[-8.5-1.0]	4.5[-18.7-22.4]
BUPFLOp	-3.9[-9.2-1.7]	0.4[-17.2-19.5]	6.0[-1.8-14.1]	-6.0[-11.7–1.1]	-4.5[-9.6-1.0]	-13.1[-65.7-29.7]	-3.9[-8.4-0.9]	4.5[-18.7-22.4]
BUPSERp	-3.0[-8.1-2.7]	0.6[-16.8-19.8]	6.4[-1.5-14.3]	-5.7[-11.6–0.6]	-4.7[-9.8-0.9]	-11.8[-64.2-31.2]	-2.6[-7.0-2.1]	4.7[-18.5-22.6]
BUPVENp	-3.5[-8.6-2.0]	0.5[-17.1-19.5]	6.2[-1.5-14.2]	-5.6[-11.3–0.7]	-4.4[-9.5-1.1]	-12.8[-65.4-30.5]	-3.3[-7.9-1.4]	4.6[-18.6-22.5]
CITESCp	-3.6[-8.8-2.1]	0.4[-17.3-19.6]	6.2[-1.7-14.3]	-5.7[-11.4–0.8]	-4.6[-9.6-0.9]	-12.7[-65.4-30.5]	-3.5[-8.0-1.1]	4.5[-18.5-22.4]
CITPARp	-3.5[-8.8-2.1]	0.4[-17.3-19.7]	6.0[-1.8-14.1]	-5.8[-11.6–0.9]	-4.6[-9.7-1.0]	-12.5[-64.9-30.6]	-3.3[-8.0-1.6]	4.6[-18.5-22.5]
CITSERp	-3.5[-8.7-2.3]	0.5[-17.2-19.6]	6.2[-1.7-14.1]	-6.0[-11.8–0.9]	-4.6[-9.7-1.0]	-12.5[-65.3-30.4]	-3.4[-8.2-1.3]	4.6[-18.6-22.6]
CITVILp	-3.6[-9.0-2.2]	0.4[-16.9-19.6]	6.1[-1.7-14.2]	-6.0[-11.6–1.2]	-4.4[-9.4-1.1]	-12.8[-65.6-30.3]	-3.4[-8.1-1.4]	4.5[-18.6-22.4]
DESDULp	-3.9[-9.2-1.6]	0.4[-17.3-19.5]	6.1[-1.7-14.0]	-6.1[-11.8–1.2]	-4.6[-9.6-0.9]	-12.8[-65.4-30.3]	-3.9[-8.5-0.8]	4.5[-18.6-22.3]
DULESCp	-4.0[-9.2-1.7]	0.4[-17.1-19.6]	6.0[-1.7-14.0]	-6.2[-12.0–1.3]	-4.6[-9.7-1.0]	-12.8[-65.3-30.3]	-4.1[-8.8-0.7]	4.5[-18.7-22.3]
DULFLOp	-4.0[-9.3-1.7]	0.4[-17.3-19.5]	6.0[-1.9-14.1]	-6.0[-11.9–1.0]	-4.6[-9.6-0.9]	-12.8[-65.6-30.2]	-3.7[-8.2-1.1]	4.5[-18.6-22.3]
DULPARp	-3.9[-9.0-1.8]	0.4[-17.0-19.6]	6.0[-1.9-14.0]	-6.1[-11.7–1.1]	-4.7[-9.7-0.8]	-12.8[-65.6-30.2]	-3.9[-8.3-0.6]	4.5[-18.6-22.4]
DULVORp	-2.9[-8.1-2.6]	0.4[-17.1-19.6]	6.4[-1.2-14.4]	-5.4[-11.1–0.6]	-4.0[-8.9-1.4]	-12.8[-65.5-30.2]	-2.3[-6.5-2.1]	4.5[-18.6-22.4]
ESCFLOp	-4.3[-9.6-1.3]	0.4[-17.3-19.5]	5.9[-1.8-13.9]	-6.3[-12.0–1.4]	-4.7[-9.8-0.9]	-12.9[-65.3-30.1]	-4.5[-9.2-0.3]	4.4[-18.7-22.3]
ESCPARp	-3.4[-8.5-2.3]	0.4[-17.2-19.6]	6.2[-1.6-14.3]	-5.7[-11.4–0.7]	-4.3[-9.2-1.1]	-12.8[-65.4-30.2]	-3.1[-7.7-1.6]	4.6[-18.5-22.6]
ESCSERp	-3.4[-8.6-2.2]	0.5[-17.0-19.7]	6.2[-1.6-14.2]	-5.8[-11.6–0.7]	-4.5[-9.5-1.0]	-12.8[-65.6-30.5]	-3.3[-8.1-1.6]	4.6[-18.6-22.5]
FLOMIRp	-4.1[-9.4-1.7]	0.4[-17.2-19.6]	6.0[-1.9-14.0]	-6.0[-11.9–0.9]	-4.5[-9.6-1.0]	-13.2[-65.9-29.8]	-3.7[-8.8-1.6]	4.5[-18.5-22.4]
FLOPARp	-3.8[-9.0-1.8]	0.4[-17.2-19.6]	6.1[-1.7-13.9]	-5.3[-10.9–0.5]	-4.7[-9.8-0.9]	-12.8[-65.3-30.3]	-5.0[-9.5–0.3]	4.5[-18.6-22.4]
FLOREBp	-3.3[-8.5-2.3]	0.5[-17.1-19.6]	6.3[-1.5-14.3]	-5.9[-11.7–0.9]	-4.8[-9.8-0.7]	-11.5[-64.1-31.4]	-3.3[-7.9-1.4]	4.5[-18.7-22.3]
FLOSERp	-3.8[-9.3-2.1]	0.4[-17.4-19.5]	6.1[-1.8-14.2]	-6.0[-11.8–0.9]	-4.5[-9.5-1.0]	-13.1[-65.9-29.8]	-3.7[-8.9-1.7]	4.5[-18.6-22.4]
FLOVENp	-4.0[-9.1-1.5]	0.4[-17.2-19.6]	6.1[-1.7-14.2]	-5.8[-11.5–0.7]	-4.8[-9.7-0.7]	-12.8[-65.6-30.3]	-3.9[-8.1-0.4]	4.5[-18.6-22.4]
FLOVILp	-3.8[-9.3-2.2]	0.4[-17.1-19.6]	6.0[-1.8-14.1]	-6.0[-11.7–0.9]	-4.7[-9.7-0.8]	-12.8[-65.5-30.4]	-3.7[-8.8-1.7]	4.5[-18.7-22.4]
MIRTRAp	-3.8[-9.3-2.1]	0.4[-17.1-19.6]	6.0[-1.8-14.1]	-5.9[-11.7–1.0]	-4.6[-9.6-1.0]	-13.0[-65.7-30.0]	-3.9[-8.6-1.0]	4.5[-18.7-22.4]
PARREBp	-4.0[-9.2-1.6]	0.4[-17.2-19.5]	6.0[-1.9-14.1]	-5.8[-11.4–1.0]	-4.6[-9.7-1.1]	-13.0[-65.7-29.9]	-3.7[-8.8-1.5]	4.5[-18.5-22.5]
PARVENp	-4.3[-9.5-1.2]	0.4[-17.1-19.6]	5.9[-1.8-14.1]	-6.1[-11.9–1.0]	-4.5[-9.5-1.0]	-13.6[-66.3-29.5]	-3.7[-9.0-1.6]	4.5[-18.7-22.5]
TRAVENp	-3.7[-8.9-2.0]	0.4[-17.2-19.5]	6.0[-1.7-14.1]	-6.0[-11.8–0.9]	-4.6[-9.6-0.9]	-13.1[-65.7-29.9]	-3.7[-8.3-1.1]	4.5[-18.6-22.4]
VENVORp	0.0[0.0-0.0]	0.0[0.0-0.0]	0.0[0.0-0.0]	0.0[0.0-0.0]	0.0[0.0-0.0]	0.0[0.0-0.0]	0.0[0.0-0.0]	0.0[0.0-0.0]

Figure S 5: Covariate pairwise comparisons. Plots illustrating pairwise comparisons between single-comparison placebos adjusted for the various covariates. Comparisons with credible intervals (CrIs) excluding zero are highlighted; blue color favors the treatment indicated in the row, and vice versa, red color favors the treatment indicated in the column.

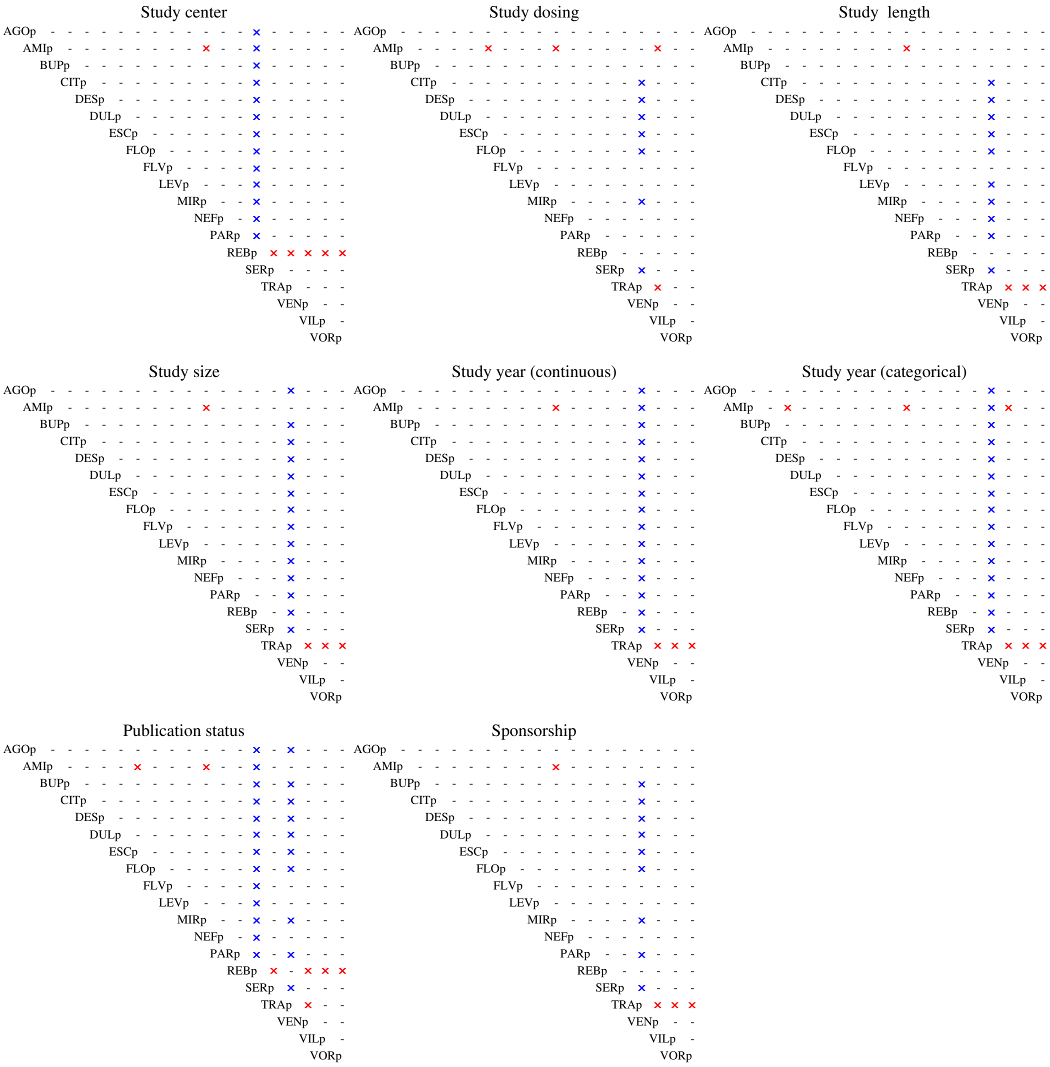

5 Code models

5.1 Code unadjusted model

```
nummodel <- function(){
  for(i in 1:ns){#LOOP THROUGH STUDIES
    tmp1[i]<-id[i]
    w[i,1] <- 0
    delta[i,1] <- 0

    #Baseline model
    mu[i] ~ dnorm(lambda[T[i]],taumu)  #model3: Two normal distribution for trial baselines #Categorical
        variable for mixture
    T[i] ~ dcat(P[])                    #Categorical variable for mixture

    for (k in 1:na[i]) {#LOOP THROUGH ARMS
      varx[i,k]<-pow(se[i,k],2)
      prec[i,k]<-1/varx[i,k]
      y[i,k]~dnorm(theta[i,k],prec[i,k])
      theta[i,k]<-mu[i]+delta[i,k]
      dev[i,k]<-(y[i,k]-theta[i,k])*(y[i,k]-theta[i,k])*prec[i,k]

    }
    sumdev[i] <- sum(dev[i,1:na[i]]) #summed residual deviance contribution for this trial
    for (k in 2:na[i]) {#LOOP THROUGH ARMS
      delta[i,k] ~ dnorm(md[i,k],taud[i,k])#trial-specific LOR distributions
      md[i,k] <- d[t[i,k]] - d[t[i,1]] + sw[i,k] #mean of LOR distributions (with multi-arm trial correction
       )
      taud[i,k] <- tau *2*(k-1)/k#precision of LOR distributions (with multi-arm trial correction)
      w[i,k] <- (delta[i,k]-((d[t[i,k]]-d[t[i,1]]))) #adjustment,multi-arm ROTS
      sw[i,k] <- sum(w[i,1:(k-1)])/(k-1) #cumulative adjustment,multi-arm trials

    }
  }

  ssumdev <- sum(sumdev[]) #Total Residual Deviance

  ##prior distribution for basic parameters
  d[1:nt] ~ dmnorm(md1[1:(nt)],prec1[1:(nt),1:(nt)])
  for (i in 1:(nt)) {md1[i]<-md0 }
  for(k in 1:(nt)){taud1[k,k]<-tau.sqd}
  for (i in 1:(nt-1)){
    for (k in (i+1):(nt)){
      taud1[i,k]<-0.5*tau.sqd
      taud1[k,i]<-taud1[i,k]
    }}
  prec1[1:(nt),1:(nt)]<-inverse(taud1[1:(nt),1:(nt)])
  md0~dnorm(0,1)
  taudx ~ dnorm(0,1)%_%I(0,)
  tau.sqd<- pow(taudx,2)


  sd ~ dunif(0,100)#vague prior for between-trial SD
  tau <- 1/pow(sd,2)#between-trial precision = (1/between-trial variance)
```

```
  sigma2.e <- 1/tau  # residual error variance
  # tausq ~  dlnorm(-2.13,2.4964)#%_%I(,0.000207)
  # sigma2.e <- 1/tausq
  # tau <- sqrt(tausq)
  # tausq ~  dlnorm(-4.28,0.3857)%_%I(,0.000207)
  # prec <- 1/tausq
  # tau <- sqrt(tausq)

  mumean ~ dnorm(0,0.0001)
  taumu<-1/pow(sdmu,2)
  sdmu~dunif(0,100)

  #priors for the flexible trial baseline model
  P[1:2] ~ ddirch(alpha[])
  eta ~ dnorm(0.0, 1.0E-3)%_%I(0.0, )
  lambda[2] <- lambda[1] +eta
  lambda[1] ~ dnorm(0.0, 0.0001)
  alpha[1] <- 1
  alpha[2] <- 1

  #pairwise ORs and LORs for all possible pair-wise comparisons
  for (c in 1:(nt-1)) {
    for (k in (c+1):nt) {
      or[c,k] <- exp(d[k] - d[c])
      lor[c,k] <- (d[k]-d[c])
    }
  }


}
```

5.2 Code covariate adjusted model

```
nummodel <- function(){
  for(i in 1:ns){#LOOP THROUGH STUDIES
    tmp1[i]<-id[i]
    w[i,1] <- 0
    delta[i,1] <- 0
    b1[i,1]<-0      #No covariate adjustment the non-intervention / control an

    #Baseline model
    mu[i] ~ dnorm(lambda[T[i]],taumu)  #model3: Two normal distribution for trial baselines
    T[i] ~ dcat(P[])                    #Categorical variable for mixture

    for (k in 1:na[i]) {#LOOP THROUGH ARMS
      varx[i,k]<-pow(se[i,k],2)
      prec[i,k]<-1/varx[i,k]
      y[i,k]~dnorm(theta[i,k],prec[i,k])
      theta[i,k]<-mu[i]+delta[i,k]
      dev[i,k]<-(y[i,k]-theta[i,k])*(y[i,k]-theta[i,k])*prec[i,k]

    }
    sumdev[i] <- sum(dev[i,1:na[i]]) #summed residual deviance contribution for this trial
    for (k in 2:na[i]) {#LOOP THROUGH ARMS
      delta[i,k] ~ dnorm(md[i,k],taud[i,k])#trial-specific LOR distributions
```

```
    md[i,k] <- d[t[i,k]] - d[t[i,1]] + sw[i,k] + b1[i,k] #mean of LOR distributions (with multi-arm trial
     correction)
    taud[i,k] <- tau *2*(k-1)/k#precision of LOR distributions (with multi-arm trial correction)
    w[i,k] <- (delta[i,k]-((d[t[i,k]]-d[t[i,1]]) + b1[i,k])) #adjustment,multi-arm ROTS
    sw[i,k] <- sum(w[i,1:(k-1)])/(k-1) #cumulative adjustment,multi-arm trials

    #baseline adjustment
    b1[i,k] <-(beta[t[i,k]]-beta[t[i,1]])*X[i]
  }
}

ssumdev <- sum(sumdev[]) #Total Residual Deviance
##prior distribution for basic parameters
d[1:nt] ~ dmnorm(md1[1:(nt)],prec1[1:(nt),1:(nt)])
for (i in 1:(nt)) {md1[i]<-md0 }
for(k in 1:(nt)){taud1[k,k]<-tau.sqd}
for (i in 1:(nt-1)){
  for (k in (i+1):(nt)){
    taud1[i,k]<-0.5*tau.sqd
    taud1[k,i]<-taud1[i,k]
  }}
prec1[1:(nt),1:(nt)]<-inverse(taud1[1:(nt),1:(nt)])
md0~dnorm(0,1)
taudx ~ dnorm(0,1)%_%I(0,)
tau.sqd<- pow(taudx,2)

beta[1] <- 0#covariate effect is zero for reference treatment
for (k in 2:nt){#LOOP THROUGH TREATMENTS
#  d[k] ~ dnorm(0,.0001)#vague priors for treatment effects
  beta[k] ~ dnorm(B,tauB)     #modelB
}
B ~ dnorm(0,.0001)#vague prior for covariate effect
tauB<-1/pow(sdB,2)
sdB~dunif(0,100)

sd ~ dunif(0,100)#vague prior for between-trial SD
tau <- 1/pow(sd,2)#between-trial precision = (1/between-trial variance)
sigma2.e <- 1/tau  # residual error variance
# tausq ~  dlnorm(-2.13,2.4964)#%_%I(,0.000207)
# sigma2.e <- 1/tausq
# tau <- sqrt(tausq)
# tausq ~  dlnorm(-4.28,0.3857)%_%I(,0.000207)
# prec <- 1/tausq
# tau <- sqrt(tausq)

mumean ~ dnorm(0,0.0001)
taumu<-1/pow(sdmu,2)
sdmu~dunif(0,100)

#priors for the flexible trial baseline model
P[1:2] ~ ddirch(alpha[])
eta ~ dnorm(0.0, 1.0E-3)%_%I(0.0, )
lambda[2] <- lambda[1] +eta
lambda[1] ~ dnorm(0.0, 0.0001)
alpha[1] <- 1
alpha[2] <- 1

for (k in 1:nt){
  for (j in 1:nz) { dz[j,k] <- d[k] + beta[k]*z[j] } #treatment effect when covariate = z[j]
}
```

```
  #pairwise ORs and LORs for all possible pair-wise comparisons
  for (c in 1:(nt-1)) {
    for (k in (c+1):nt) {
      #when covariate is zero
      or[c,k] <- exp(d[k] - d[c])
      lor[c,k] <- (d[k]-d[c])
      #at covariate=z[j]
      for (j in 1:nz) {
        orz[j,c,k] <- exp(dz[j,k] - dz[j,c])
        lorz[j,c,k] <- (dz[j,k]-dz[j,c])
      }
    }
  }

}
```

References

[1] Cipriani. “Comparative Efficacy and Acceptability of 21 Antidepressant Drugs for the Acute Treatment of Adults with Major Depressive Disorder: A Systematic Review and Network Meta-Analysis”. *The Lancet* 391, 10128 (2018), pp. 1357–1366.

[2] Furukawa, Cipriani, Atkinson, Leucht, Ogawa, Takeshima, Hayasaka, Chaimani, and Salanti. “Placebo Response Rates in Antidepressant Trials: A Systematic Review of Published and Unpublished Double-Blind Randomised Controlled Studies”. *The Lancet Psychiatry* 3, 11 (2016), pp. 1059–1066.
